# Effects of Metal Oxide Nanoparticles in Zebrafish

**DOI:** 10.1155/2022/3313016

**Published:** 2022-02-04

**Authors:** Marta d'Amora, Tiziana Julia Nadjeschda Schmidt, Soultana Konstantinidou, Vittoria Raffa, Francesco De Angelis, Francesco Tantussi

**Affiliations:** ^1^Istituto Italiano di Tecnologia, Via Morego 30, 16163 Genova, Italy; ^2^Department of Biology, University of Pisa, S.S. 12 Abetone e Brennero 4, 56127 Pisa, Italy

## Abstract

Metal oxide nanoparticles (MO NPs) are increasingly employed in many fields with a wide range of applications from industries to drug delivery. Due to their semiconducting properties, metal oxide nanoparticles are commonly used in the manufacturing of several commercial products available in the market, including cosmetics, food additives, textile, paint, and antibacterial ointments. The use of metallic oxide nanoparticles for medical and cosmetic purposes leads to unavoidable human exposure, requiring a proper knowledge of their potentially harmful effects. This review offers a comprehensive overview of the possible toxicity of metallic oxide nanoparticles in zebrafish during both adulthood and growth stages, with an emphasis on the role of oxidative stress.

## 1. Introduction

The field of engineered nanomaterials has gained increasing attention over the last years in human health science, optoelectronics, agriculture, food science, and in everyday use products [1]. Metal oxide nanoparticles (MO NPs) have shown fascinating physical and chemical properties, such as good sensitivity, catalytic and selective activity, unusual adsorptive behavior, and superparamagnetic state (Table 1) [2, 3]. Different studies focused on easy and efficient synthesis methods, a few of which implementing “green chemistry approaches,” providing thus a variety of different strategies to efficiently achieve the desired size, shape, structure, morphology, stabilization, and nonagglomeration. One of the most important advantages of MO NPs is the ease of their surface modification allowing for the functionalization of numerous molecules to improve their stability and biocompatibility [4]. Hence, MO NPs serve as a promising tool for biomedical applications. Metal oxide nanoparticles are known for their antimicrobial properties [5, 6] and cytotoxic effects [2]. The synthesis method of the nanoparticles plays a critical role in determining their properties, i.e., their biological and optical characteristics. For instance, it seems that the smaller the nanoparticles are, the higher is the antibacterial activity they exert [2, 7]. Moreover, due to their metallic core, MO NPs can be used as plasmon resonance agents, in cancer therapeutics and theranostics (Table 1) [3, 8].

Different classes of MO NPs are exploited in commercially available daily life products and biomedical applications (Table 1). The most commonly applied ones correspond to three types of MO NPs, the titanium dioxide (TiO_2_), iron oxide (IO), and zinc oxide (ZnO) nanoparticles. The TiO_2_ and ZnO nanoparticles are extensively used in sunscreens due to their ability to attenuate UV radiation and as antimicrobial reagents given their antibacterial properties. On the other hand, IO NPs are employed in several medical applications, such as hyperthermia-based anticancer therapy and iron-deficient anemia treatment, as well as in magnetic resonance imaging (MRI).

The rising demand and use of nanotechnologies inevitably questions their impact on the environment. In this framework, TiO_2_, IO NPs, and ZnO nanoparticles could be released i.e., via bathing and cause any toxic effects in the aquatic habitats [9]. Since metal oxide nanoparticles are exposed to humans and are extensively used in daily life and industrial content, their ecotoxicological profile should be evaluated [1]. Metal oxide nanoparticles present some toxic defects as they [10] internalize in the cells and interact with the DNA, proteins, and organelles. Here, they can induce the formation of reactive oxidative species (ROS) and interfere with the antioxidant mechanisms. The excessive production of ROS, and accumulation in cells and tissues, leads to oxidative stress and subsequently to lipid peroxidation, DNA damage, inflammation, and cell death [11]. Undoubtedly, this along with the penetration abilities of the nanoparticles enhances their toxic effects in the cells [2]. The ROS usually include singlet oxygen (^1^O_2_), hydroxyl radical (·OH), and superoxide radical (O_2_·^−^) [12]. The excess of ROS can be detected by missregulation of antioxidant enzymes, either of their genes or of their activity. In this context, the expression and activity of superoxide dismutase (SOD), catalase (CAT), glutathione peroxidase (GP), and glutathione S-transferase (GST) are most commonly evaluated. SOD catalyses the disproportionation of superoxide anions (O_2-_) into oxygen (O_2_) and hydrogen peroxide (H_2_O_2_), and CAT and GP reduce the hydrogen peroxide levels. GST instead plays a role in detoxification by removing glutathione. Their normal regulation is critical for the survival of the cells. On the other hand, to prevent the imbalance between production and catalysis of ROS, various cytoprotective genes might be influenced. Nuclear factor erythroid 2-related factor 2 (Nrf2) is a transcription factor with such a protective antioxidative role, targeting numerous redox cycling enzymes, including the ones named before [13]. Upon transcription of Nfr2 target genes, the ROS level is normalized, leading to detoxification and the reestablishment of homeostasis.

One of the main factors believed to be responsible of the MO NP-induced toxicity is the release of their appropriate metal ions and the ions' inherent toxic effects in the cells [11].

Hence, there is a not only a great need to fully understand the mechanisms underlying nanotoxicity but also to develop innovative strategies allowing to mitigate this effect in order to fully exploit their potential [9] for our purposes. Most of the toxicological profiles of metal oxide nanoparticles have been studied *in vitro*, in suspensions of MO NPs, in several cell types, and *in vivo* in different invertebrates and vertebrate animal models. Zebrafish (*Danio rerio*) represent a link between the *in vitro* cell culture studies and *in vivo* animal models. The zebrafish embryo toxicity test (ZET) known also as the early life stage test (ELS) is widely accepted as a valid model system for the evaluation of ecotoxicological effects and as a preclinical *in vivo* model [14, 15]. Zebrafish emerged as a model for *in vivo* toxicity screening of nanoparticles due to several characteristics [16]. First of all, zebrafish and humans are highly genetically conserved. Additionally, zebrafish grow rapidly and are transparent during early life stages, two very important characteristics that allow studying easily the development. A variety of developmental endpoints have been already described to evaluate toxicity during the embryonic stages, including the hatching timing, pericardial and yolk sac edema, spinal curvatures, tail malformations, swim bladder abnormalities, and mortality rates [4, 17, 18]. These phenotypes, together with other characteristic ones, are visible and detectable up to the first five days after fertilization of the embryos, allowing thus zebrafish serve for fast screenings. Due to the small size of these fish, and the high number of embryos that they produce, different parameters can be tested simultaneously. The standardization of tests using zebrafish to assess adverse effects induced by nanomaterials allows gathering reproducible and reliable results. Doing so would allow counteracting contradictory results obtained in the past, implementing other model systems while introducing a controllable amount of bias in the experimental setups. For instance, as it is evident in this review, it is critical to test not only the metal oxide nanoparticles but also the appropriate metal ions they release. This is necessary to estimate the direct contribution of the dissolved ions in the establishment of toxicity and to identify the potentially involved mechanisms [9].

**Figure 1:**
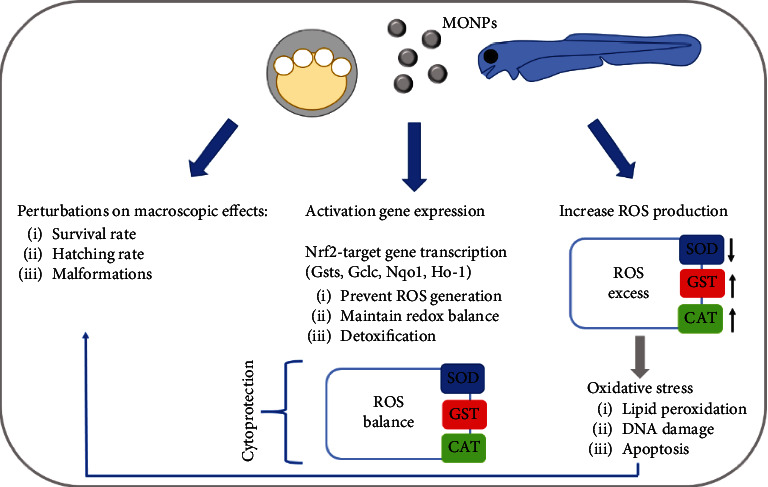
Overview of the MO NP effects of in zebrafish, with an emphasis on oxidative stress.

In this framework, we focus on the toxicity studies performed on zebrafish embryos and adult zebrafish, stating the effects of three different types of metal oxide nanoparticles: titanium dioxide (TiO_2_), iron oxide (IO), and zinc oxide (ZnO) nanoparticles. Different studies using either one type or a combination of these analyzed the toxicokinetic behavior of the particles on the development of zebrafish and/or adult zebrafish. This review will provide thus an extended overview of the impact of metal oxide nanoparticle exposure on zebrafish while conferring a better understanding of the potentially underlying toxicity mechanisms, such as the induction of oxidative stress and apoptosis in *Danio rerio (*Figure 1).

## 2. Titanium Dioxide Nanoparticles

Titanium dioxide nanoparticles are one of the most commonly employed manufactured nanoparticles in a wide range of applications, including building materials [21], medical treatments [22], and personal care and food products [23]. Titanium oxide and zinc oxide are considered as “GRAS” (generally recognized as safe) by the US Food and Drug Administration (FDA) and by the International Agency for Research on Cancer [24]. TiO_2_ is highly stable, biocompatible, and a semiconductor material. This increasing interest and use of TiO_2_ in our daily life and several applications are due to their fascinating properties, such as good optical performance, electrical characteristics, durability, and corrosion resistance [25] [26–28]. In addition, since TiO_2_ NPs are excellent photocatalysts, they can produce peroxide under ultraviolet (UV) illumination. Indeed, they are extensively used in photocatalytic applications [24]. TiO_2_ NPs are nontoxic, and due to their optical and UV absorption properties, they are used in sunscreens, though there are more restrictions in the EU than in the United States (EUR-Lex -32020R0217 - EN - EUR-Lex). One of the main biomedical applications of metal oxide nanoparticles is their use as drug carriers [24]. For instance, TiO_2_ NPs were functionalized with daunorubicin (DNR), an anticancer drug, for controllable release of the drug by lowering the pH from 7.4 to 5. In this way, the side effects of DNR could be reduced, and the cytotoxicity of cancer cells augmented due to the improved penetration of the drug in the cell [29]. Another example showing the anticancer activities of TiO_2_ NPs comes from the work of Masoudi et al. who prepared TiO_2_ NPs with doxorubicin hydrochloride (DOX) to induce cytotoxicity [30]. In addition, as other MO NPs, TiO_2_ NPs are used in tissue engineering and in antibacterial applications [31]. TiO_2_ are also used as biosensors, such as in nanowires, to recognize bacteria *Listeria monocytogenes i*n food with high specificity [32]. Metal oxide nanoparticles can induce the production of reactive oxidative stress, an important characteristic employed for cancer cytotoxicity. Considering all the above-mentioned applications in medicine, it was critical to validate that the produced reactive oxygen species levels are nontoxic [33]. Anyway, the wide use of TiO_2_ NPs led to their inevitable release in the aquatic environments, arising harmful threats for ecosystems and living organisms. For this reason, the adverse effects of TiO_2_ NPs need to be considered and evaluated. In the past years, different toxicity studies have elucidated the in vitro and in vivo behavior of TiO_2_ NPs and their biointeractions with several cell lines and animal models. In particular, several works have assessed the potential harmful effects of TiO_2_ NPs both in embryos and in adults (Table 2).

### 2.1. Effects of TiO_2_ NPs during the Development

The first research studies of titanium dioxide biointeraction with zebrafish have reported their nontoxicity [34–36]. Zhu et al. have assessed the impact of titanium dioxide nanoparticles on zebrafish growth, reporting that the treatment of embryos with high doses (up to 500 mg/L) of TiO_2_ NPs did not lead to a significant decrease of the survival rate or delay in the hatching rate or presence of morphological abnormalities [34, 37]. However, larvae treated with low doses of nanoparticles presented behavioral alterations at 120 hours post fertilization (hpf). At doses of 0.1, 0.5, and 1 mg/L TiO_2_ NPs, larvae had a significantly lower velocity and higher activity level compared to the samples of control, while higher concentrations of 5 and 10 mg/L did not show any changes. These perturbations can be attributed to physiological injuries or neurotoxicity induced by TiO_2_ NP treatment [37]. Moreover, this type of nonlinear concentration-reaction relationship was already previously shown [38, 39]. This nonmonotonic behavior could be related to superimposition of linear concentration interaction of constituent biological counterbalances. Previous works in different aquatic species have shown that rainbow trout [40] and carp [41] treated with TiO_2_ NPs had gill injuries, including fusion and hyperplasia in filaments and lamellae and edema. These damages can implicate a reduction of oxygen assumption ability and alter the activity. On the other hand, both treated fish species presented oxidative stress in the brain [40, 41] that could cause neurotoxic effects [42]. To evaluate the potential implication of oxidative stress in the perturbations noted in TiO_2_ NP-exposed zebrafish, the embryos were cotreated with an antioxidant compound, NAC, and/or an antioxidant suppressor, the inhibitor of GSH synthesis, the buthionine sulfoximine (BSO) [37]. The used doses of BSO and NAC were 5 and 50 *μ*M, respectively, while the selected concentration of TiO_2_ was one of those implicated in behavioral changes (1 mg/L). The cotreatment did not lead to perturbations on the hatching or survival rate or malformations. Moreover, NAC or BSO did not modify the behavioral perturbations induced by titanium dioxide nanoparticles. This observation indicated that, as well as oxidative stress, other processes can be implicated. The mentioned research studies indicated the nontoxicity of the tested TiO_2_ NPs. Nevertheless, the potential of titanium dioxide nanoparticles to generate reactive oxygen species under illumination indicates that they can induce adverse effects in a photo-dependent manner. Moreover, the consequent oxidative stress can lead to lipids, proteins, or DNA injuries and ultimately to cell death [43, 44]. To verify this assumption, Bar-Ilan et al. treated the zebrafish embryos with different doses of TiO_2_ NPs under a metal halide light [45]. First, a solution of TiO_2_ NPs illuminated under this source generates an important amount of ROS. The survival rate of treated and illuminated zebrafish with TiO_2_ NPs showed a lethal dose of 300 *μ*g/mL, while the embryos that were exposed to nanoparticles but not illuminated had a value superior to 1000 *μ*g/mL. By increasing the exposure time to 8 days, all the illuminated larvae died at a dose of 100 *μ*g/mL. The combined exposure of light and nanoparticles led also to different malformations, affecting prevalently the head, tail, yolk, and heart [45]. Moreover, the ROS generation led by TiO_2_ NPs in the treated embryos and larvae was demonstrated by using the dihydroethidium (DHE), an *in vivo* fluorescent superoxide indicator. Samples treated with TiO_2_ NPs and illuminated presented fluorescence, reporting the presence of ROS. Moreover, the use of a transgenic zebrafish, Tg (are: eGFP), enables the observation of the oxidative stress response directly in the zebrafish. In particular, a dose of TiO_2_ NPs ≤ 1000 *μ*g/mL under illumination generates DNA damage. The same authors treated the zebrafish embryos with different concentrations (0.01 to 10 000 ng/mL) of two different batches of titanium dioxide nanoparticles for a longer temporal window (over 23 days post fertilization, dpf) to detect subsequent and increasing effects due to ROS generation, such as damages to macromolecules [46]. A significant mortality rate was observed for all the tested doses in comparison to control samples. In normal conditions, a certain number of zebrafish do not survive during the metamorphosis period, when they are especially vulnerable. Exposure to light and TiO_2_ NPs speeded up the death of fish in this life stage. In addition, the nanoparticles induced distinctive abnormalities and perturbations in the growth. The embryos and larvae showed reduced size, not developed fin rays, deformations of craniofacial structures, and absence or abnormal organization in the pigmentation. The larvae treated with 1000 *μ*g/mL presented a swim bladder with only a single lobe. Moreover, an important increase of 8-hydroxy-2′-deoxyguanosine (8-OHdG) detected by ELISA revealed an indication of oxidative stress and intracellular damages. Another study explored the effects of size on the biointeractions of citrate-functionalized TiO_2_ NPs on zebrafish during the development under illumination [47]. Zebrafish were exposed to 6, 12, or 15 nm sizes to citrate-TiO_2_ NPs for 120 hpf. The smallest NPs (6 nm) were the ones that presented the highest dose-dependent harmful effects, with a LC50 value of 23 *μ*g/mL; the LC50 for 12 and 15 nm NPs were 610 *μ*g/mL and not detectable, respectively. Moreover, the exposed larvae showed several phenotypic abnormalities, including the opaque yolk, axial curvatures, craniofacial defects, yolk sac, and pericardial edema. On the other hand, high levels of hydroxyl radical (˙OH) and ROS were detected by using specific indicators, the 3′-(*p*-aminophenyl) fluorescein (APF) and the acetyl ester of 5-(and 6-) chloromethyl-2′,7′-dichlorodihydrofuorescein diacetate (CM-H2DCFDA). The detected values were higher for 6 nm NPs than those for the 12 and 15 nm NPs [47].

Jovanovic et al. evaluated the potential neuroimmunological effects of TiO_2_ NPs injected together with hydroxylated fullerenes in the otic vesicle of zebrafish [48]. To this end, the expression of different genes linked to the immune and nervous systems was analyzed. The coinjection caused the downregulation of three clusters of genes, associated with the circadian rhythm, transport, vesicular trafficking, and immune response.

Due to the concomitant presence in the aquatic environment of toxicants and nanoparticles, their combined effects were tested in zebrafish. In particular, three different studies evaluated the combined effects of pentachlorophenol (PCP), or deca-BDE (BDE-209), or bisphenol A with titanium dioxide nanoparticles, assessing a possible effect impacting on *Danio rerio* growth [49, 50]. The study on the effects of PCP and TiO_2_ NPs focused mainly on the genotoxicity and oxidative stress evaluation [49]. The values of survival and hatching rates in samples treated with both PCP and PCP plus nanoparticles were similar, while the incidence of malformations was higher in the coexposed zebrafish larvae exposed. Regarding oxidative stress, zebrafish treated only with nanoparticles presented an alteration in glutathione content, SOD activity, and malondialdehyde, while no increase in ROS production was revealed in comparison to the control groups. However, coexposure to PCP and nanoparticles to fish led to a decrease in the SOD activity and GSH content when compared to the sample treated with PCP alone. Moreover, coexposure led to an increase in ROS production and important levels of MDA in comparison to the single treatment. Similarly, the coexposure caused an important upregulation of two genes, implicated in the glutathione metabolism and oxidative damage, *sod1* and *nrf2* [49]. These findings indicate that titanium oxide nanoparticles enhance the PCP metabolism, causing genotoxicity and oxidative stress in zebrafish during their development. In another study, the effects of BDE or BDE plus nanoparticles were investigated in the embryos for 7 dpf [50] with emphasis on the neurodevelopment and thyroid tissues. The survival and hatching rates of the samples treated with BDE or BDE plus nanoparticles had similar values over 90% for both the biological parameters. Since a previous work reported a thyroid endocrine disruption led by BDE-209 [50], the values of TH were noted. Samples cotreated with BDE and nanoparticles led to an important increase in the thyroxine (T4) values in comparison to the ones exposed only to the toxicant. No difference in the triiodothyroxine (T3) levels was found. The analysis of different genes implicated in TH regulation, and metabolism reported an important upregulation in the expression of the thyroglobulin (*tg*), thyroid-stimulating hormone*β* (*tshβ*), and iodothyronine deiodinase 2 (*dio2)* genes, in the cotreated larvae. The same control was performed on genes implicated in zebrafish neurodevelopment. Downregulations of *α1-tubulin* and methyl-CpG-binding domain (*mbd)* genes were detected, while the expression of the growth-associated protein 43 (*gap-43)* genes was normal in cotreated fish. In accordance with these results, also the expression of the *mbd* protein was perturbed, while the one of *α1-tubulin* was not affected. Coexposed larvae presented also a reduction in the swimming speed [50]. These findings reported that TiO_2_ NPs enhance the metabolism of BDE. In addition, the exposure of zebrafish to BDE plus nanoparticles led to neurodevelopmental toxicity and thyroid endocrine perturbation. This study together with the one performed by Fang et al. demonstrated that TiO_2_ NPs can absorb toxicants, suggesting that toxicity assessments on contaminates should take into account also the copresence of titanium dioxide nanoparticles. The same research groups investigated the impact of TiO_2_ NPs on neurogenesis with emphasis on the retina in a parallel study [51]. The embryos treated with 1 mg/L of TiO_2_ NPs until 72 hpf showed a normal phenotype, with no increase in the mortality rate or significant incidence of malformations. In addition, the expression of the atonal homolog 7 (*atho7*) in the retina of treated fish was found to be similar to the control by using the *in situ* hybridization. Moreover, the expression of different cell types was investigated through immunostaining (Zn12, Zpr1, and Zpr3 antibodies), which allowed to further investigate neuronal differentiation. At 3 dpf, all the components of the retina (cones, ganglion cells, and rods) were well-differentiated in all the samples, demonstrating the absence of TiO_2_ NP-induced effects on the neurogenesis [51]. Finally, the analysis of microglia migration revealed the absence of perturbations in macrophage migration in the retina and the brain of the treated larvae. Another work evaluated the toxicological profile of bisphenol A (4,4′-isopropylidenediphenol, BPA) and TiO_2_ NPs [52]. Fish treated with only TiO_2_ NPs showed a normal survival rate and presented no important malformations compared to the controls. On the other hand, after treatment with up to 40 mg/L of TiO_2_ NPs, the hatching rate was importantly decreased. The combined exposure to BPA and TiO_2_ NPs led to a significant dose-dependent decrease of the survival rate and induced different malformations in the larvae, such as spine deformation, weak pigmentation, and pericardial edema. These abnormalities were much more intense in the cotreated zebrafish compared to ones treated only with BPA. These findings, as in the case of the previously analyzed toxicants, demonstrated that the effect of a chemical is enhanced by the presence of TiO_2_ NPs. The combined impact, in all three cases (PCP, BDE, and BPA), caused a potentiation of the harmful effects.

Another study evaluated the toxicity of TiO_2_ NPs in combination with humic acid (HA) [53]. The presence of HA led to a change in the survival rate of embryos. Indeed, the survival rate of eggs treated only with TiO_2_ NPs was 85% and increased to 95% in the presence of HA. This indicated that the presence of HA mitigates the harmful effects exerted by TiO_2_ NPs.

In the same year, Faria et al. evaluated the oxidative effects of three different titanium dioxide nanoparticle aggregates (NM TiO_2_) in the presence or absence of solar irradiation [54]. These aggregates were different in terms of crystal structure or coating: NM-103 and NM-104 (89% TiO_2_, primary crystal size of 20 nm), P25 (99.5% TiO_2_ and a primary size of 21 nm), and microsized TiO_2_ (98.5% TiO_2_ nontreated surface). The three aggregates did not affect the survival and hatching rates of the treated larvae nor induce significant abnormalities in the embryos/larvae. Only one dose of microsized TiO_2_ caused a shortening of the length of the larvae. In addition to a general decrease in SOD activity, glutathione levels were perturbed. However, the analysis of photo-oxidative stress indicated that the P25 NPs produced aggregates that led to the highest levels of reactive oxygen species in comparison to the other NM TiO_2_. Taken together, titanium dioxide nanoparticle aggregates did not cause strong toxicity or mortality to zebrafish during the development.

Another study assessed the potential toxicity of TiO_2_ NPs produced with a particular technique, using the high-energy ball milling (HEBM) for 15 h, in comparison to the bulk particles [55]. The determined value of LC50 was 90 *μ*g/mL similar to the one of bulk NPs (95 *μ*g/mL). Surprisingly, the TiO_2_ NP exposure enhanced the hatching rate of embryos. The embryos and larvae presented some abnormalities (both body and organs). Finally, the analysis of ROS showed lower levels for the TiO_2_ NPs produced by the HEBM method, compared to the bulk one. The same research group focused their attention again on TiO_2_ NPs produced using the HEBM method by milling bulk TiO_2_ particles for different times (5, 10, and 15 h). The survival rates and hatching rates of exposed embryos significantly decreased or increased, respectively, in a dose-dependent manner. In both cases, the strongest effect was found for embryos/larvae treated with TiO_2_ NPs milled for the longest period (15 h). As varying the milling times allows modifying the size and the charge of NPs, it was possible to assess potential effects induced by these alterations. In particular, the evaluated biological parameter was found to be dependent on the NP milling time. Moreover, by using an *in vivo* and *in silico* computational approach, steatosis, apoptosis, and oxidative stress were assessed. Surprisingly, 5 h, 10 h, and 15 h milled TiO_2_ NPs led to ROS quenching. This particular behavior of industrial TiO_2_ NPs could be probably due to the production of oxygen vacancies during the HEBM approach. In addition, the analysis of perturbations in neutral lipids allows determining the TiO_2_ NP-induced steatosis. Zebrafish treated with TiO_2_ NPs showed a concentration-dependent accumulation of lipid in different areas of the animal, including the tail, the head, and the notochord. Moreover, acridine orange staining revealed a high number of apoptotic cells in the tail and head of samples treated with TiO_2_ NPs. Different computational investigations were performed to reveal the interaction of NPs with the *sod1* gene, implicated in the ROS production, or the *apoa1a61* (apo-lipoprotein), or docking the tumor protein 53 (*tp53)* protein (apoptotic factor) with TiO_2_ NPs. These analyses allow understanding the key role of lipid accumulation and ROS quenching in the TiO_2_ NPs toxicity in zebrafish during the development. In particular, the production of TiO_2_ NPs via the HEBM approach led to a change not only in the zeta potential and size of the synthetized nanoparticles but importantly in the oxygen vacancies, causing harmful effects. Moreover, the alteration of the activity of *sod1* causes a perturbation of *tp53*. The final pathway caused lipid alterations, apoptosis, and oxidative stress [56].

Even if different studies have already analyzed the effects of TiO_2_ NPs on the most common toxicological endpoints (hatching, survival rates, and abnormalities), a deeper study was performed to evaluate the hatching rate at different time points (34, 58, 82, 106, and 130 hpf) by exposure of embryos to different TiO_2_ NPs doses (0.01,10, and 1000 mg/mL) [57]. The 73% of embryos exposed to the highest dose of TiO_2_ NPs hatched prematurely between 34 and 58 hours post exposure (hpe) in comparison to the control group, exposed only to normal medium (58-82 hpe). This indicates that the presence of TiO_2_ NPs can induce premature hatching of the embryos.

The impact of ultrasmall TiO_2_ NPs (USNPs) (1-3 nm, 10, 100, and 1000 mg/L) with a focus on vascular toxicity was studied in zebrafish during development [58]. Simple soaking exposure to the highest concentration of TiO_2_ USNPs (1000 mg/L) induced 100% mortality and together with the intermediate dose (100 mg/L) delayed hatching. No vascular effects were noted at 120 hpf. On the other hand, embryos injected a 0 hpf with TiO_2_ USNPs (1 ng/embryo) presented several malformations such as aneurysm and pericardial edema, while the ones injected in the circulatory systems at 48 hpf did not present any perturbations or vascular toxicity. To assess the specific impact on angiogenesis, eggs were treated by soaking or injected with 100 mg/L of TiO_2_ USNPs. In both cases, nanoparticles led to a reduction in length of the growing intersegmental vessels (ISVs). To comprehend the mechanism related to the impact on angiogenesis, the expression of genes involved in vascular toxicity was evaluated. Only the expression of Myosin IC (*Myo1c)*, involved in glomerular development, was affected by TiO_2_ USNPs. These data demonstrated for the first time the vascular effects of ultrasmall TiO_2_ on zebrafish during development.

The effects of coating and size on the toxicity of TiO_2_ NPs were evaluated by exposure of embryos to nanoparticles with different sizes (4, 10, 30, and 134 nm) prepared at 6 different concentrations (50, 500, 5000, 50000, and 250000 *μ*g/L) [59]. TiO_2_ NPs of 4 and 30 nm did not exert toxicity on zebrafish, while the 10 nm and 134 nm had a low impact on the mortality rate at 5000 and 250000 *μ*g/L, respectively. Moreover, embryos treated with different sized NPs, and the respective doses presented no necrotic cells or only a low amount of them. The expression of metalloprotein 2 (Mt2) by *in situ* hybridization was found to be comparable to the control samples. These findings were in line with previous studies, reporting the absence or low toxicity of TiO_2_ NPs.

Also, the specific possible neurotoxicity of TiO_2_ NPs was evaluated in zebrafish during the development [60]. The treated embryos/larva did not present an alteration in the survival rate in comparison to the control samples. Contrarily, the hatching rate was decreased, and a significant incidence of abnormalities (tail flexure and pericardial edema) at 96 hpf was observed. In addition, the behavior of larvae was affected by the treatment with nanoparticles, with a decrease in the total distance of swimming of the larvae at 96 hpf compared to the controls. This indicates a toxic effect of TiO_2_ NPs, but without consequent mortality. TEM images showed that once internalized in the embryos, TiO_2_ NPs can cross the blood-brain barrier (BBB) and localize in the larvae brain. A high ROS production with consequent oxidative stress was detected in the treated larvae. On the other hand, histological analysis showed high apoptosis levels in the hypothalamus. Moreover, the analysis of the genes alpha-synuclein (*α-syn*), *parkin*, ubiquitin C-terminal hydrolase L1 (*uchl1m*), and *pink1*, implicated in the Lewy body formations, revealed their upregulation [60]. Finally, zebrafish larvae presented a decrease in the dopaminergic neurons. All these findings underline that TiO_2_ NPs induced effects that are similar to symptoms of Parkinson's disease (PD).

Since TiO_2_ NP can interface with heavy metals in the aquatic environment, few studies have assessed the effects on zebrafish of TiO_2_ NPs and Pb cotreatments [61, 62]. To this end, zebrafish embryos were coexposed to TiO_2_ NPs (0.1 mg/L) and several doses of Pb (0, 5, 10, 20, and 30 *μ*g/mL) [61]. The hatching and survival rates were similar in the samples that were cotreated or exposed only to one of the two compounds. However, adverse effects on organogenesis were revealed only in the coexposed larvae. To evaluate the potential impact of the cotreatment on the thyroid endocrine system, the levels of T3 and T4 were determined. A decrease in the T3 and T4 levels was observed when zebrafish were treated with 30 *μ*g/mL of Pb alone or with all the doses of Pb plus TiO_2_ NPs. On the other hand, no changes were found in the embryos exposed only to NPs. In addition, the expressions of *tg* gene and transthyretin (*TTR*) gene were found to be downregulated, while the one of the thyroid-stimulating hormone (*tsβ*) resulted to be upregulated, in treatments relying on both the compounds. Moreover, the genes sonic hedgehog protein A precursor (*shha*), *gfap*, *α-tubulin*, and *mbp*, implicated in the development of the central nervous system (CNS), were downregulated in comparison to the samples exposed only to different doses of Pb. Finally, larvae coexposed presented a significant decrease in the swimming speed. All these perturbations indicated that TiO_2_ NPs could induce toxicity in the thyroid endocrine system and the development of the zebrafish CNS [61]. In a similar study, embryos were treated with Pb or Pb plus TiO_2_ NP for 2 days with a subsequent depuration (144 h) [62]. The uptake and complex formation between TiO_2_ NPs and Pb were assessed by transmission electron microscopy-energy dispersive spectrometry (TEM-EDS). The survival and hatching rates of treated embryos/larvae were up to 85% for all the investigated cases. A significant perturbation in the two biological parameters was observed only in the case of 40 *μ*g/L Pb plus TiO_2_ NPs when compared to the samples treated with Pb alone. Moreover, the coexposure led also to a reduction in the larval swimming speed. This perturbation in the locomotor behavior is in line with the previous finding of Miao et al. Further, the expressions of genes implicated in brain formation and development and, specifically, those encoding for glial fibrillary acidic protein (*gfap*), HuC (*elavl3*), and synapsin IIa (*syn2a*) were evaluated. A downregulation was observed in the expression of all three genes. These results indicated that the presence of TiO_2_ NPs could enhance the neurotoxicity effects of Pb in zebrafish during the development [62].

A recent study has deeply assessed the specific and potential neurotoxic effects of TiO_2_ NPs on *Danio rerio* [63]. Zebrafish were treated until 6 dpf with 4 different doses of nanoparticles (0.01, 0.1, and 1.0 mg/L nano-TiO_2_ and 1.0 mg/L micro-TiO_2_). The survival and hatching rates were not affected by any exposure, while the body weight and length of larvae were decreased at 1.0 mg/L nano-TiO_2_ as well as rotation times and the swimming speed. The treatment of the transgenic line Tg (HuC-GFP) and Tg (hb9-GFP) with nano-TiO_2_ caused, respectively, perturbation in the neurogenesis and the motor neuron axon length. Similarly, the expression of genes *α1-tubulin*, *mbp*, and *gap43* implicated in the axonal growth, and the genes *nrd* and *elavl3*, involved in the neurogenesis, were perturbed. It can be thus concluded that nano-TiO_2_ induce neurotoxic effects in zebrafish, particularly in the motor neuron axonal growth and neuronal development.

Tang et al. assessed the toxicity in embryos treated chronically with high doses of TiO_2_ NPs (100 mg/L) [64]. However, no significant changes were observed in the hatching, survival, or deformity rates.

Only one work evaluated the effects of a coating of the TiO_2_ NPs on zebrafish during development [65]. Eggs were exposed to bare TiO_2_ NPs or TiO_2_ NPs with polyelectrolyte on the surface under illumination or darkness. In particular, nanoparticles were coated with poly(sodium 4-styrene sulfonate) sodium salt (PSS, anionic) (TiO_2_ NPs/PSS,) and polyallylamine hydrochloride (cationic, PAH) (TiO_2_ NPs/PSS/PAH). No changes in the survival rate were observed for all the treated samples under both conditions. In addition, the gene expression of peroxisomal membrane protein 2 (*Pxmp2*), a marker of hypoxia, hypoxia-inducible factor 1 (*HIF1)*, a marker for membrane function, and *SOD2*, a marker of oxidative stress, of samples exposed to bare TiO_2_ NPs, TiO_2_ NPs/PSS, and TiO_2_ NPs/PSS/PAH were measured. The level of *SOD2* mRNA resulted to be perturbed, under both illumination and dark conditions for all the different treatments. *Pxmp2* expression was normal in all the cases. The mRNA level of *HIF1* presented a significant alteration only when the experiments were performed under illumination. These findings demonstrated that the toxicity of TiO_2_ NPs can be influenced by several factors, including the presence/absence of illumination and surface coating.

### 2.2. Effects of Titanium Dioxide Nanoparticles on Adults

Studies on the effects of titanium dioxide nanoparticles on adult zebrafish are limited. Griffit et al. treated adult zebrafish females with titanium dioxide nanoparticles and analyzed their possible effect on gills in terms of both morphological changes and perturbations in gene patterns. Titanium dioxide NPs did not alter significantly the gill histopathology after 24 and 48 h of treatment [35]. Moreover, the investigation of transcriptional activity revealed important changes in the expression of 171 genes after 48 h of treatment, with 111 genes downregulated and 60 upregulated. Interestingly, some of these genes are implicated in the function of ribosomes [35].

**Figure 2:**
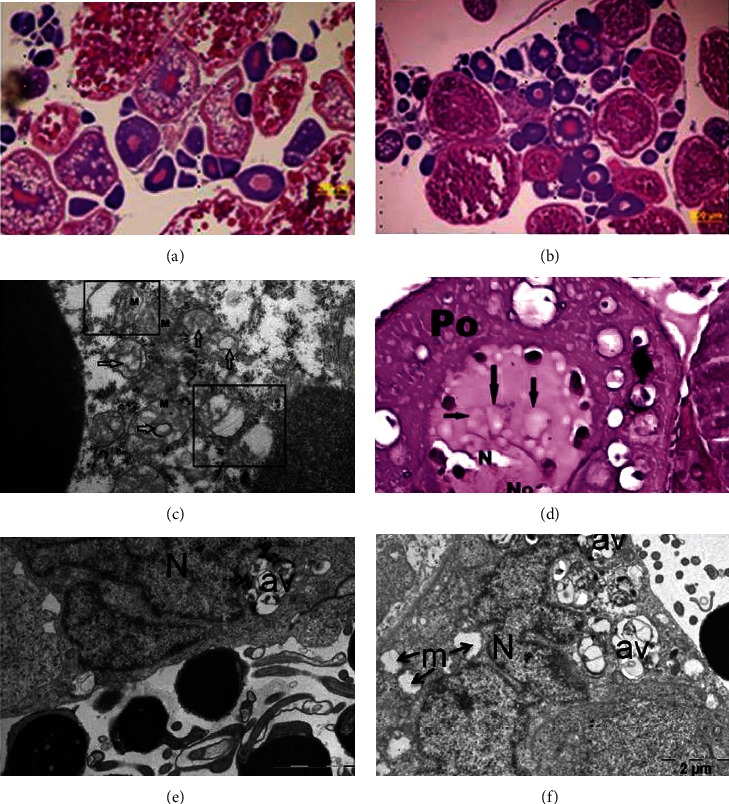
Images of (a–d) ovaries and (e–f) testis tissues of adult zebrafish treated with TiO_2_ NPs. Reproduced with permissions from [66, 69, 70].

In 2011, Wang et al. performed a prolonged (91 days) and chronic treatment of zebrafish with titanium oxide nanoparticles, focusing on the potential impact on reproduction [66]. After 9 weeks, females treated with TiO_2_ NPs started to produce a decreased number of eggs. In addition, the mortality rate of embryos produced by exposed females presented an increase in the mortality rate at 2 dpf. This observation indicates that prolonged treatment with TiO_2_ NPs impairs the survival and reproduction of zebrafish. Since the decreased number of eggs generated by females can be linked to a problem in folliculogenesis, histological analysis of the ovaries was performed. TiO_2_ NPs caused a perturbation in the follicular stages, reporting a block in the development probably due to nanoparticles interacting with the follicles (Figure 2). The gene expression implicated in the development of oocytes was evaluated by a microarray of ovarian tissues. 0.1 and 1 mg/mL of TiO_2_ NPs led to an important alteration of several genes (1043 downregulated/2383 and 471 upregulated/2069), demonstrating a perturbation in the functionality and maturation of the ovary [66].

Ramsden and his research group investigated the biointeractions of TiO_2_ NPs on zebrafish of 14 dpf, focusing on the reproduction and different physiological parameters, such as organ anatomy, hematology, and osmoregulation [67]. The treated adult did not present behavioral abnormalities or mortality. As the number of white blood cells changed only on the last day of exposure (14) for all the tested doses of TiO_2_ NPs, this observation can be neglected. The amounts of trace metals and whole electrolytes were normal for all the temporal windows of investigation. Also, Na^+^K^+^-ATPase activities in the liver, gills, and brain were found similar to the control, demonstrating good osmoregulation. However, the values of GSH in the same tissues were higher in the treated adults in comparison with the control ones. The histological analysis of all these tissues did not reveal any significant changes. Indeed, no aneurisms or edema were detected in the gills, together with no parenchymatic changes in the liver. The morphological structures of the brain and the gonads resulted to be normal. The lack of damage revealed via the histological analysis in the investigated tissues suggested the absence of intracellular oxidative damage [67]. These results are in agreement with the previous study conducted by Chen et al. [37].

The toxicological profile of two different formulations of TiO_2_ NPs (anatase, TA or an anatase/rutile mixture, TM, form) on zebrafish was assessed under different illumination settings (visible light or visible and ultraviolet light) [68]. No mortality was detected in embryos treated with TA between 4 and 72 hpf in all the investigated samples. Five percent mortality was present only after 96 hpf in the group treated with 100 mg/L of TA under UV light. On the other hand, zebrafish treated with TA under UV illumination showed a lower hatching rate as well as shortening in terms of larval body length. Treatment with TM led to egg coagulation and perturbation of the larval equilibrium in all the samples, while the survival and hatching rates were significantly decreased and increased, respectively, only under UV light. Under UV illumination, the analysis of biochemical markers revealed a decrease in the enzymatic activity of acid phosphatase, GST, and CAT [68]. These changes indicated a state of oxidative stress.

Akbulut et al. focused their research on the potential and specific effects of TiO_2_ NPs on ovaries. To this end, adults were treated for 5 days with different doses of nanoparticles (1, 2, and 4 mg/L). The analysis was performed by using both histological staining (hematoxylin and eosin on paraffin sections) and TEM [69]. Several toxic effects of TiO_2_ NPs were observed in the ovaries. Treated samples presented structural changes and degeneration of the follicles. In particular, several vacuolizations in the cytoplasm indicated evident forms of specific cell death (paraptosis, type III). Further, the tissue showed mitochondrial vesiculation and chromatin condensation (Figure 2). In addition to this, mitochondria presented swelling and mitotic catastrophe. Hence, TiO_2_ NPs led to paraptosis in adult zebrafish and inhibited oogenesis. These findings are in line with the previous work of Wang et al. related to zebrafish development [66] in which they reported perturbations in the female reproduction, with evident defects in folliculogenesis.

The impact of TiO_2_ NPs on testis was further investigated by treatment of zebrafish with 1 mg/L, 2 mg/L, and 4 mg/L of nanoparticles, subsequent dissection, and fixation of the testis, and final analysis of sections by TEM [70]. TiO_2_ NPs affected the testis in a dose-dependent manner, causing swelling and loss of cristae and degenerated mitochondria in spermatocytes and Sertoli cells (Figure 2). Zebrafish exposed to TiO_2_ NPs presented a high amount of necrotic cells. As the TiO_2_ NP-induced alterations in the Sertoli cells caused damage in the testicular morphology, a concomitant possible negative impact on fertility cannot be excluded.

To assess potential genotoxic effects induced by TiO_2_ NPs, adult zebrafish were exposed to NP doses similar to the one present in the aquatic environment (1 and 10 *μ*g/L) for different time points (5, 7, 14, 21, and 28 days) [71]. The genotoxicity was investigated by using three different and complementary approaches. First, the level of DNA damage was evaluated using the comet assay. A significant percentage of DNA fragmentation in treated zebrafish was detected at a dose of 10 *μ*g/L of TiO_2_ NPs at 5 days while reaching a maximum after 14 days in comparison to controls. In addition, the number of apoptotic cells in zebrafish exposed to the same dose of nanoparticles detected by diffusion assay was found to be significantly reduced after 10 days of treatment, supporting the results obtained with the comet assay. Moreover, the DNA injuries were further analyzed by the RAPD-PCR technique. This analysis showed a clear deviation from the control in terms of DNA band pattern of adults exposed to TiO_2_ NPs for 14 and 21 days, even if after 28 days this observation was partially mitigated. The same technique showed that the genome stability (GTS%) decreased notably at 14 days but then recovered partially after 28 days. These data demonstrate clearly that the highest tested concentration (10 *μ*g/L) of TiO_2_ NPs caused genotoxic effects in adult zebrafish after 14 and 21 days of exposure [71].

Tang et al. assessed the toxicity of TiO_2_ NPs both in embryos and adults. Here, they focused their attention on the potential impact of NPs on the liver, gills, and intestine with emphasis on oxidative stress [64]. The activities of GSTs, CAT, and SOD were investigated in adults treated with different doses of nanoparticles. The enzymatic activity of all three investigated proteins was shown to be significantly decreased when compared to controls. Especially in the gills and liver, these alterations are associated with the induction of a condition of oxidative stress. Moreover, no important perturbations of their activities were detected in the intestine. This observation could be attributed to the low absorption of TiO_2_ NPs in the small intestine after ingestion. On the other hand, the expression of CAT, SOD, and GST genes was upregulated in all the investigated organs. It can be concluded that although TiO_2_ NPs induce upregulation of genes involved in the antioxidant machinery, the corresponding level of traduced proteins was not sufficient to counteract the production of ROS, causing thus oxidative stress in the liver and gill of adult zebrafish.

As in the case of zebrafish during the development, also adult fish were exposed both to TiO_2_ NPs (100 *μ*g/L) and BPA (0, 2, and 20 *μ*g/L) or their mixture for 90 days, to understand the possible effects on the gut microbiota [72]. The cotreatment of TiO_2_ NPs and BPA caused a change in the intestinal microbial community. In addition, the impact of TiO_2_ NPs on zebrafish development and in particular on the intestine (oxidative stress and inflammation) was found to be dose- and sex-dependent. The oxidative responses due to the cotreatment were linked to a different amount of *Lawsonia* and *Hyphomicrobium*. The treatment with a mixture of TiO_2_ NPs and BPA had an impact on the gut microbiota, with consequent effects on the *Danio rerio* as a host organism.

A subsequent work evaluated the mortality and injury induced by TiO_2_ NPs (5 and 40 mg/L) [73]. The TiO_2_ NP exposure was connected with an increase of both bacteria in the water and animal motility. Moreover, the increase in bacteria was found in the gut and not in the caudal and dorsal fins. *Actinobacteria*, *Bacteroidetes*, and *Proteobacteria* were found to be the main component of the flora of the gut, containing a high amount of bacteria present in zebrafish treated with TiO_2_ NPs. These findings suggest a correlation between the zebrafish mortality caused by TiO_2_ NPs and bacterial infections.

## 3. Iron Oxide Nanoparticles (IO NPs)

Iron oxide nanoparticles (IO NPs) can be designed with a wide range of physicochemical and biological properties, making them a useful platform for biological and medical applications. Due to their versatile characteristics, colloidal stability, and increased biocompatibility and degradability, they have been intensively studied and implemented in clinics over the past decades. As iron oxide is a naturally occurring mineral, it allows for ecofriendly nanoparticle (NPs) synthesis, without the need to rely on potentially toxic chemical procedures and costly reagents. Moreover, it is responsible for the inherent magnetic properties that characterize these kinds of nanoparticles [74]. While IO NPs come in different shapes and sizes, generally, they share a basic design, composed of a magnetic iron core (mostly magnetite, maghemite, or *γ*-Fe_2_O_3_ for biological and environmental applications) comprised of one or multiple crystals [15, 75]. Based on this crystalline core, IO NPs can be grouped into three main categories: micron-sized magnetic iron oxide particles (MP IO), superparamagnetic particles (SP IO) displaying a hydrodynamic diameter larger than 50 nm, and ultrasmall ones (USP IO), with less than 50 nm [76]. It follows that the response of IO NPs to an external magnetic field (MF) is influenced by their composition, as well as by their dimension [5]. Indeed, the coating does not only prevent the IO NPs from aggregating and protects them from environmental influences but importantly confers the basis for the attachment of other biomolecules creating a plethora of opportunities for possible applications [77]. Notably, IO NPs are so far the only class of metallic nanoparticles that have been approved for clinical use, i.e., in cancer bioimaging, hyperthermia-based therapy, and the treatment of iron deficiency [78]. As the particles' size and surface coating influence strongly its biodistribution, several medical applications have been developed accordingly [79]. Polymeric coated-IO NPs are clinically proven and widely approved as magnetic resonance agents, where they can be further implemented to display dysfunctional processes [76, 79, 80]. Iron oxide-based nanoparticles hold thus great promise in theranostic applications and are extensively exploited as targeted drug delivery systems due to their capability to undergo versatile functionalization processes [80, 81]. As IO NPs are generally of hydrophobic nature, a coating of hydrophilic layers does not only improve their biocompatibility but allows further for subsequent attachment of biological molecules of interest [81, 82]. Here, a prominent example is given by anemia therapy where IO NPs have been successfully used as a remedy for many years [80, 82]. In contrast, in cancer treatment, IO NPs have been implemented as in vivo cytotoxicity and apoptosis inducers, leading to a significant reduction of the number of malignant cells [74, 78]. Indeed, IO NPs are the only nanoparticles approved for hyperthermia-based therapy in humans, an approach that exploits their magnetic properties for the generation of heat when exposed to an alternating magnetic field (AMF) [78, 83]. A completely different approach is instead based on a similar concept: it has been shown that the application of an MF to IO NP-labeled neuronal cells allows to stimulate them mechanically, inducing consequently a cellular response, transduced, e.g., in neurite outgrowth [84–86]. Achieving controllable magnetic guidance of neurons could contribute importantly to the understanding of neurodegenerative diseases and their treatment as knowledge in this field of research is scarce. Indeed, recently several studies tried to take advantage of the unique properties of IO NPs for tissue engineering and regenerative medicine [82]. In addition, IO NPs can act as potent catalysts, due to their particular physiochemical properties that cannot be found in their bulk counterparts, and as a consequence, they have been implemented successfully to address plenty of different economic and environmental issues [87].

As demonstrated by their wide range of clinical applications, IO NPs display without any doubt a high degree of safety in living organisms. However, most studies assessing the cytotoxicity of IO NPs are performed in cell culture model systems and lack in whole animal systems [86]. While *in vitro* studies generally revealed an absence of toxicity, it must be kept in mind that nanoparticles can be still identified as invading non-self-components by the immune system of living organisms. Here, they could trigger immunogenetic responses, such as an allergic reaction, hypersensitivity, localized or systemic inflammation, immunosuppression, or a combination of all [75, 78]. The size, shape, and surface coating, but also the administration method, the exposure condition, and the host itself, play a role in the induction of a potentially unexpected health effect to the IO NPs [15, 88, 89]. The type of interaction the IO NPs establish with the immune system depends highly on their characteristics as these govern ultimately their biodistribution in the organisms. Indeed, several IO NP-based contrast agents have been withdrawn from the market in several countries after causing adverse side effects [72]. In this context, it is particularly noteworthy that several studies revealed the immune reaction to IO NPs being either immune-stimulating or immune-suppressive [78, 89]. Especially when present in a high concentration, IO NPs can favor the outcome of toxic side effects [90].

Over the past years, IO NPs have further gained growing attention in commercial and industrial applications while increasing consequently also their release in the environment [15, 91]. Here, a particular concern is given to the aquatic environment, as alarming estimations regarding extensive sedimentary depositions of these nanopollutants demand an accurate evaluation of their ecotoxicological impact on this niche, and thus ultimately on human health [15]. As a consequence of the globally increasing implementation of IO NPs in several branches, the deposition of these particles in various life domains is inevitable. Several studies have thus focused on the adverse effects induced by IO NPs on aquatic organisms [14], with particular focus on zebrafish. Different studies have elucidated the possible harmful effects of IO NPs both during the development and in adult organisms (Table 3).

### 3.1. Effects of IO NPs on Zebrafish during Development

Although IO NPs have been widely accepted as nontoxic, care must be taken as different studies reveal contradictory results [92]. In general, it must be distinguished between primary and secondary IO NP-dependent induced toxicity. The latter one is given, e.g., by the induction of an inflammatory status in response to the entry of NPs in the organism with subsequent activation of several downstream responses, such as an increase in systemic levels of reactive oxygen species (ROS). A primary response instead requires the intracellular localization of the NPs and involves the responses that take place at a cellular level [88]. Several studies showed that NPs can interfere with the chorion by blocking its pores, limiting thus the exchange of nutrients and oxygen. However, especially this factor is strongly influenced by the size of the IO NPs and their concentration [15, 89]. Usually, small IO NPs can pass the chorion without any disturbance and do not induce any embryo toxicity if not exceeding in concentration. In addition, the thickness of the chorion can be altered due to NPs sticking to it and accumulating on its inner/outer surface, especially when present in high concentrations [15]. Together with the accumulation of NPs on its surface, this could lead to a retard of embryo growth and/or altered hatching due to hypoxia and the establishment of ROS [15, 89]. In the study performed by Pereira and colleagues, no deviation from normal hatching behavior was observed for any of the investigated doses, exposure conditions, and iron forms, indicating that the treatments did not exhibit any adverse effect during the early stages of development and presumably did not interfere negatively with the embryonic gene expression and/or chorionic surface [15]. Being hatching the transition point from the developing embryo to the free-living larvae, it is often evaluated in toxicity tests, as it allows to assess the overall developmental status [75]. However, a deviation of the hatching time point cannot be strictly associated with toxicity, as the hatched larvae might not display signs of underdevelopment during later life stages. Nevertheless, the absence of an alteration of hatching behavior can be indicative of the fact that the IO NPs that have surpassed the chorion accumulate in the organs, displaying their potential toxic effects only at later time points of the zebrafish development. Indeed, IO NPs functionalized with citrate and their dissolved counterpart revealed a mild embryo toxic effect after an exposure period of 144 h [15]. Nevertheless, the absence of an alteration of hatching behavior can be indicative of the fact. However, this effect was shown to be dose- and exposure-type dependent. While the treatment of the larvae with *γ*-Fe_2_O_3_ NPs resulted in a high mortality rate after 144 h when surpassing a certain concentration, the lethal dose was diverse according to the exposure type. In this context, static exposure (0.6-10 mg/L) appeared to be less toxic, as higher concentrations were needed for the induction of death when compared to the semistatic setup (0.3-10 mg/L) [15]. Regarding the iron ions, no difference was observed in terms of the type of exposure but resulted in a high mortality rate in all groups treated with doses >0.3 mg/L. These observations hint towards the fact that the exposure conditions of the IO NPs need to be considered in the establishment of nanotoxicity and that the induced effect is partially independent of the presence of iron ions themselves. This comes as static exposure of IO NPs enables for treatment with a higher dose without an increase in mortality when compared to the same concentration of free iron. A slightly different trend was observed with regard to neurotoxicity, assessed by the SCF, a common marker for the potential neurotoxicity of substances. While the SCF of embryos treated with IO NPs under static and semistatic conditions did not reveal any neurotoxicity, this was not true for their dissolved counterpart. Here, only semistatic exposure to iron ions (5 mg/L) led to a reduction in the spontaneous embryo contraction, when compared to lower doses (0.3, 0.6 mg/L) and the control group. The increase of toxic effects caused by the free iron ions when compared to the same concentration of IO NPs strengthens the assumption that proper surface coating can reduce the potentially adverse effects of IO NPs. This hypothesis is in line with the finding that the coated *γ*-Fe_2_O_3_ NPs induced a low frequency of morphological alterations on zebrafish larvae and embryos in comparison to their dissolved counterpart under static conditions [15]. However, this was not true for semistatic exposure, where a large number of malformations were observed for both iron forms. In addition, the investigated concentrations of IO NPs did not alter the morphometric parameters of zebrafish exposed for 144 under static and semistatic conditions. Higher doses of free iron ions (1.25 and 2.5 mg/L) induced instead notable physiological alterations (i.e., reduction of the area of the swim bladder, yolk sac, head height, and body length) under semistatic exposure. Nevertheless, by implementing uncoated *γ*-Fe_2_O_3_ NPs, this effect was reversed, with the embryos displaying pericardial edema, tissue ulceration, and spinal curvature [15]. Taken together, the degree of toxicity induced by IO NPs is not only strongly influenced by the physicochemical composition of the surface of the NPs but also by the exposure condition itself that could potentiate the adverse effect notably.

Another recent study revealed a dose-dependent delay of embryo hatching after incubation of 96 h with Congo red-labeled Fe_3_O_4_ (Cr@Fe_3_O_4_). The observed effect was both dose- (>200 *μ*g/mL) and time-dependent. In particular, the highest dose of 800 *μ*g/mL induced a reduction of around 70% in the overall hatching behavior. However, as no adverse effect in terms of hatching was revealed for the bare NPs at any investigated concentration, it might be concluded that the toxic impact is due to the presence of the dye [93]. Indeed, another possibly cytotoxic effect to take into consideration for IO NPs is the one given by their surface structure. This is further demonstrated by the fact that proper surface coating of the IO NPs could ameliorate the observed adverse effects to a certain degree underlying again the importance of the physicochemical composition of nanoparticles in the induction of toxicity and teratogenicity [89]. Indeed, it has been shown that specific coatings, such as dextran or polyethylene, can significantly reduce the toxicity of IO NPs for a wide range of concentrations [77, 94].

To investigate further this aspect, Oliveira et al. reported the effect of different coatings in the elicitation of SP ION-induced toxicity [77]. In addition to the classic sublethal endpoints, they assessed also behavioral patterns after 5 days of exposure (locomotion, thigmotaxis, and escape response). They evaluated the impact of different coatings: dextran (SP ION-DX), chitosan (SP ION-CS), carboxy-silane (SP ION-T), polyethylene glycol (SP ION-T-PEG), and silica (SP ION@SiO_2_). The animals were evaluated daily for mortality, hatching rate, and malformations using a stereomicroscope. Interestingly, only SP ION-CS led to a reduction in the survival of zebrafish embryos when administered in concentrations of >2 mM. More in detail, the deadly effect was dose-dependent and was accentuated continuously after 2 days of exposure, leading to a 100% mortality rate after 5 days. As reported in other studies, precipitation of high concentrated NPs might be at the basis of this adverse effect [14, 77, 95].

Next, the hatching behavior was evaluated between 48 and 72 hpf. According to the specific surface coating, slight differences were revealed. In line with the increased mortality rate, animals treated with 8 mM of SP ION-CS died even before hatching. While 2 mM of SP ION-CS and SP ION@SiO_2_ delayed the hatching, all other groups led to mild premature hatching at all investigated concentrations. The fact that none of the investigated IO NPs induced any morphological malformations after 5 days of incubation, even at the highest investigated dose, supports the idea that appropriate surface coating can favor the biocompatibility of IO NPs when compared to the same dose of bare NPs [14]. However, as the animals treated with high concentrations of SP ION-CS deceased before analysis could take place, no conclusion about teratogenicity in this context could be given. With regard to the behavioral evaluation, similar to what has been observed for the hatching rate, only SP ION-CS (0.125 mM) and SP ION@SiO_2_ 82 mM) revealed a deviation in their locomotor activity in comparison to the controls—although these results were not completely convincing. For all investigated particles, an anxiogenic effect could be excluded. However, in terms of the escape response, zebrafish treated with SP ION-CS, SP ION-T-PEG, and SP ION@SiO_2_ showed a significant decrease in their performance [77]. The study performed by Oliveira et al. strongly affirms that surface coatings do mitigate potentially toxic effects of IO NPs by reducing their reactivity while increasing their colloidal stability. Nevertheless, this effect might be bilateral. Indeed, as demonstrated by Jurewicz et al., while IO NPs functionalized with a certain compound did not impact negatively the zebrafish development, the same compound induced toxicity when administered alone at the corresponding concentration [93]. In this context, a previous study evaluated the effect of pure chitosan nanoparticles on zebrafish embryos [96]. Here, the authors showed that chitosan induced mortality and hatching delay in a dose-dependent manner, together with the induction of morphological alterations. At the basis of this toxicity were increased levels of ROS, apoptosis, and physiological stress [96]. These findings could corroborate the hypothesis that together with the characteristics of the nanoparticle, the surface coating must be evaluated carefully in the assessment of potential toxicity, generated probably by a combination of both components.

To further increase the biocompatibility, Hafiz et al. synthetized IO NPs based on a green chemistry approach based on spinach. Zebrafish embryos and larvae were exposed to these 150-200 nm sized crystalline Fe_2_O_3_ particles at different concentrations for several time points, from 8 to 168 hpf. While concentrations ranging from 1 to 5 mg/L did not reveal any toxicity, higher doses of 50 and 100 mg/L had a deleterious effect on the embryos (100% mortality), with an LC50 of 10 mg/L, concomitant with a delay in hatching. The most sensitive stadium of development was identified to be 24 hpf, corresponding to the time point of organogenesis [97]. Indeed, as reported in previous studies, probably also in this case, high concentrations of IO NPs favor their aggregation, especially in the case of pristine particles due to their intrinsic reduced stability [77]. The identified toxic effects are probably a consequence of the obstruction of the pores of the chorion by the presence of IO NPs. However, the observed toxicity in the early stages of the development is mostly attributable to altered gas exchange between the embryo and its environment rather than to the IO NP composition itself [97]. Usually, IO NPs of smaller dimensions are considered more toxic than their bigger counterparts [98]. This is because smaller NPs present a bigger reactive surface area and thus could generate theoretically more ROS. In addition, smaller NPs are generally degraded more rapidly, leading to fast iron accumulation [94].

Zhu et al. evaluated the effect of the exposure of different doses of uncoated Fe_2_O_3_ NPs (0.1, 0.5, 1, 5, 10, 50, and 100 mg/L) on the early development of zebrafish [14]. For this purpose, they analyzed the embryos and larvae at different time points (6, 12, 24, 36, 48, 60, 72, 84, 96, 120, 144, and 168 h) via microscopy, emphasizing the survival, hatching rate, and morphological malformations. First, they demonstrated a correlation between IO NP concentration and hatching rate, where doses >10 mg/L induced hatching retardation and severe toxicity [14]. Indeed, the high concentration-induced increased adherence of IO NPs to the chorion is known to alter not only its thickness but also its physiological functions, as reported by other studies. The disturbance of the homeostasis of this important barrier, and especially the alteration of gas exchanges, could lead to ROS accumulation and thus ultimately to the observed developmental toxicity [14]. In particular, the establishment of hypoxia is tightly associated with the onset of oxidative stress [98]. Moreover, Zhu et al. showed that similar concentrations to the ones investigated by Hafiz et al. (0.1-10 mg/L) of naked Fe_2_O_3_ NPs did not exhibit any toxicity to embryos or larvae, while again higher concentrations reduced significantly viability (75% 50 mg/L and 45% 100 mg/L) after 168 hpf, with an LC50 corresponding to 53.35 mg/L. The survival of the embryos dropped importantly after 48 hpf, from 90% to 25% at 168 hpf, indicating that also here the embryo toxic effect exerted by the NPs is not only depending on the dose but also on the exposure time itself [14, 15, 97]. Indeed, treatment period is a crucial point in the establishment of toxicity of metal oxide nanoparticles in aquatic organisms [95] Moreover, serious malformations, such as pericardial edema, tissue ulceration, and body deformation, were observed for doses >50 mg/L. These effects were even more accentuated for the group treated with 100 mg/L [14]. In extreme cases, the treated embryos were unable to hatch and died consequently. It must be kept in mind that the observed aggregate formation and precipitation of the naked IO NPs after adding them to the maintenance medium, and which is due to their colloidal instability (especially observed for doses >10 mg/L), possibly affects the effective concentration at which the embryos/larvae have been exposed [14, 94]. However, as zebrafish embryos/larvae are demersal, this effect might be less important than previously stated, and the observed severe toxicity might be due to the IO NPs strong adherence to the organisms surface concomitant with a localized increase of released iron [14, 77, 97, 98]. Another important factor that contributes importantly to IO NPs' toxicity is the state of the iron of the NP itself; Fe^3+^ present in Fe3O4 NPs revealed a greater toxicity than Fe^2+^ stemming from Fe_2_O_3_ NPs [94]. For example, in the lung cancer cell line A549, bare Fe_2_O_3_ NPs (20-60 nm) did not reveal any toxicity for concentrations under 200 *μ*g/mL [99]. Interestingly, while bare Fe_2_O_3_ have been shown to be completely cleared by zebrafish after a prolonged exposure time, Fe_3_O_4_ particles are still retained in the organism after the same time span [95]. This observation might indicate that while the first ones are excreted through the digestive system, the latter ones may reach other organs where they then accumulate.

Once internalized by an organism, many studies have described the capture of IO NPs by cells belonging to the immune system, and their subsequent degradation concomitant with the release of iron ions [78, 88]. However, under physiological conditions, tissue macrophages are the ones in charge of replenishing the organisms' iron needs by clearance of senescent erythrocytes [100]. Under physiological conditions, free iron ions are sequestered under the form of the redox-inactive Fe^3+^, while only a small fraction used in the cellular metabolism is available as the redox-active Fe^2+^ [94]. Upon cell absorption, IO NPs accumulate usually inside lysosomes or endosomes where they are metabolized, causing the release of iron ions [94]. Although iron is involved in important biochemical processes, its intracellular levels must be monitored carefully [100, 101]. It is thus clear that any disturbance of the iron homeostasis might affect cellular functions adversely. Importantly, it has been shown that the accumulation of iron in living organisms is associated with the establishment of oxidative stress and pathological conditions [75, 102]. This is because when the iron storing ability is exceeded, the free ions lead to the production of reactive oxygen species and/or reactive nitrogen species (RNS) [15, 103]. Interestingly, while 2.5 mg/L of pure iron ions induced a 100% mortality rate, this could not be observed for the same concentration of investigated IO NPs. Generally speaking, the *γ*-Fe_2_O_3_ NPs showed low toxicity when compared to their dissolved counterpart [15]. In biological systems, the Fenton or the Haber-Weiss reaction is at the basis of the generation of ROS molecules [100, 104]. Iron is a transition metal and consequently it can change easily its valence, providing or accepting an electron [98]. Fe^2+^ reacts with hydrogen peroxide under the release of radical OH [94]. It follows that the accumulation of iron, and thus Fe^2+^, leads to the production of ROS inside the cytoplasm, which then could cause oxidative injury in cells. This would be true especially for uncoated IO NPs, which once endocytosed would release ions easily and thus elevate the intracellular iron concentration disturbing redox homeostasis [105]. Indeed, Pereira et al. showed that semistatic exposure to iron induced neurotoxicity in zebrafish embryos after 24 hours of incubation, while intact IO NPs corresponding to the same concentration did not induce this effect [15]. This comes as “increased free iron” favors the production of the highly reactive hydroxyl radicle, promoting lipid peroxidation, which in turn is further amplified in a self-sustained loop of cytotoxic events as it reacts directly with iron ions [101]. However, ROS is a normal byproduct of cellular metabolism, and as it carries a key role as an intracellular signaling molecule, its concentration is usually regulated by the antioxidative machinery of the cell [106]. Specific enzymes involved in this process ensure the maintenance of intracellular redox homeostasis [87]. It is known that exposure to xenobiotics increases the production of ROS. Under certain conditions, such as IO NP-induced reduction of the mRNA levels of genes involved in the antioxidant defense system or the direct inhibition of their activity [89], ROS can though accumulate and exert its toxicity, damaging biomolecules and the DNA, leading eventually to cell death [87, 100]. Moreover, in this context, a concomitant iron accumulation inside cells has been associated with cellular death due to oxidative injury [94]. As programmed cell death is induced in response to adverse stimuli occurring during embryogenic development, the onset of malformations is a clear indication of toxicity. It has been reported that the increase of intracellular iron levels is correlated with the dose of administered IO NPs [94], explaining thus the aggravation of the toxic effects in the cited studies implementing higher concentrations.

Several studies revealed that increasing concentrations of IO NPs induce in zebrafish larvae mainly cardiotoxic effects, such as pericardial edema, bradycardia, and cardiac blood accumulation [14, 15, 98]. IO NPs and iron accumulate preferentially in the heart as they display a high affinity for this organ, where they are known to induce several myocardial deficits as shown in mammalian model systems [15]. The cardiotoxic effects, observable in zebrafish embryos and/or larvae after incubation with IO NPs starting from a few hours post-fertilization, could be associated with the failure of the exposed cells to maintain normal physiological functions [15, 104]. Pereira et al. showed that citrate-coated *γ*-Fe_2_O_3_ NPs and iron ions reduced significantly the heartbeat rate after 48 h of semistatic exposure, with the latter ones inducing mortality when present at the highest dose (10 mg/L). Static exposure to *γ*-Fe_2_O_3_ instead did not induce any effect when compared to the control groups. However, both exposure conditions to *γ*-Fe_2_O_3_ and iron induced an increase of embryos displaying bradycardia. Higher concentrations of free iron ions (5 and 10 mg/L) resulted to be toxic for the embryos under both exposure conditions. Interestingly the semistatic exposure had an overall negative effect nearly for all investigated conditions, highlighting the importance of the surrounding circumstances in the establishment of toxicity [15, 75]. The cardiotoxic effects observed by Pereira et al. and induced by these treatments should be related to the accumulation of iron ions in this organ, known to be able to induce inflammation, lipid peroxidation (LPO), and oxidative stress, associated with tissue degeneration and cell death. More in detail, iron accumulation in cardiomyocytes induces the production of the highly toxic hydroxyl radicals [15]. Consequently, it is not surprising that the administration of free iron ions induced a much more severe effect on the treated embryos when compared to the IO NPs, although both iron forms display cardiotoxicity.

In addition, two recent studies focused on the sublethal effects induced by low concentrations of maghemite NPs [77, 98]. Similar to what has been reported previously, higher amounts of IO NPs correlate directly with hatching delay and the induction of cardiac dysfunction [98]. The heart is the first organ to function during embryogenesis, and cardiogenesis itself is one of the most sensitive processes taking place during development. In addition, after uptake, IO NPs are rapidly targeted to this organ thanks to blood circulation. It follows that environmental pollutants to which an organism might be exposed during early life stages would reveal their potentially hazardous effect, especially on this organ, leading to cardiac defects in later life. In zebrafish, cardiogenesis initiates 5 hpf [107], the time point at which most of the conducted experiments regarding IO NP exposure start. The most common targets implemented for the assessment of cardiotoxicity induced by a compound in zebrafish are usually pericardial edema and altered heart rate, the first indicating a general status of dysfunction, the latter instead pointing towards a defective cardiac function [107].

A comparable observation, which could be in line with this, was made by another research group [103]. Most of the research coping with IO NP-associated toxicity identified ROS as the main player involved [81]. However, contrary to what has been previously thought, the production of free radicals is rather attributable to reactions that take place at the surface of the IO NPs than to the dissolved iron oxide ions. Voinov et al. demonstrated that the catalytic centers present on the surface of uncoated *γ*-Fe_2_O_3_ nanoparticles are strongly responsible for the production of hydroxyl radicals [105].

Thirumurthi and colleagues described in a recent study that increased amounts of Fe_3_O_4_ nanoparticles induced malformations by triggering several pathways that are activated by the accumulation of ROS in tissues and organs [98]. To understand better the underlying phenomena, they first treated zebrafish embryos statically with several concentrations of bare IO NPs (10, 20, 40, 60, 80, 100, 120, and 140 ppm) for 96 h. After individuating the LC50 = 60.17 ppm, they choose to assess only the sublethal doses of 40 and 60 ppm in further experiments. However, the obtained results are in sharp contrast with another study performed by Malhotra et al., in which concentrations up to 1000 ppm of bare Fe_2_O_3_ NPs did not induce any mortality in exposed embryos after 96 h exposure [92]. Thirumurthi et al. monitored the animals throughout the experimental setup for survival, hatching rate, and signs of teratogenicity via microscopy. To evaluate the potential impact of iron levels in the establishment of adverse effects, they further measured the iron content in the groups using inductively coupled plasma mass spectrometry (ICP-MS) and SEM and assessed the number of dissolved iron ions in the medium and animals with atomic absorption spectroscopy (AAS). As the authors were interested in the potential underlying mechanisms triggered by the naked IO NPs, they focused their attention on important biomarkers involved in oxidative stress. Among these, alteration of the activities of acetylcholinesterase (AChE), responsible for cholinergic transmission, and Na^+^K^+^-ATPase, involved in osmoregulation, are warning bells for xenobiotic toxicity. In addition, apoptosis, ROS, and NO levels, along with LPO and protein carbonyls, hallmarks for protein oxidation were assessed among the groups. As other studies showed that IO NPs could have an impact on the antioxidative response of the cell, the authors investigated further the status of important antioxidant markers. As described in previous studies, the authors revealed a dose- and time-dependent lethality and delay in hatching (10% 40 ppm and 38% 60 ppm), concomitant with colloidal instability of the bare IO NPs associable to high doses. Precipitation of aggregates of IO NPs to the bottom of the plate leads to an immediately increased interaction with the embryos, while adhesion to the chorion and interference with the pores is known to limit oxygenation. Regarding later life stages, it was shown that 40 and 60 ppm led to a 20% and 40% augmentation in teratogenicity, respectively. In line with other studies, both investigated doses induced a reduction in the heartbeat rate (24% 40 ppm and 36% 60 ppm). As Na^+^K^+^-ATPase in combination with ROS is known to be involved in cardiotoxicity, they now focused on the activity of this enzyme. They revealed a significant dose-dependent decrease in the activity of the Na^+^K^+^-ATPase. The opposite effect was obtained for AChE, in which protein levels were increased in the treated groups. The altered activity of this enzyme is associated with developmental neurotoxicity. In line with these alterations, Thirumurthi et al. showed that upon treatment with increasing doses of Fe_3_O_4_, the animals displayed a concomitant increase of ROS, LPO, PC, and NO levels. Consequent to the augmentation of oxidants, larvae treated with 40 and 60 ppm revealed an elevated number of apoptotic bodies when compared to the control group. Due to these first results, the group focused subsequently on the antioxidant machinery, whose IO NP-induced alteration could be connected to the observed effects. Strikingly, a substantial decrease of SOD, CAT, and glutathione peroxidase (Gpx) activity was assessed in a dose-dependent manner, explaining thus the failure of the cell to counteract the oxidative stress. As described earlier, the accumulation of iron ions is thought to be involved in the establishment of oxidative stress. For this reason, the authors evaluated the amount of iron in the treated larvae. Indeed, it was shown that the exposed animals presented a concentration-dependent increase in iron. Taken together, the authors showed clearly that static exposure of zebrafish larvae for 96 hpf with Fe_2_O_3_ NPs leads to a significant increase of oxidative stress concomitant with a downregulation of the antioxidant machinery [98].

This observation was partially supported by a study conducted in another aquatic organism, the microalgae *Coelastrella terrestris* [108]. Here, they demonstrated that prolonged incubation of uncoated iron oxide NPs did reduce in a dose-dependent manner (up to 50 mg/L) viability and growth. They identified oxidative stress as a key element responsible for inducing the observed phenotype. It has been suggested that in response to the temporary increase of intracellular ROS levels induced by IO NPs, the cellular antioxidative machinery is activated, including SOD, to reduce its harmful production [106]. In line with this, SOD levels were augmented in cells of *Coelastrella terrestris* accumulating IO NPs.

Jurewicz et al. investigated further the effect of different concentrations of naked and fluorescently labeled Fe_3_O_4_ nanoparticles (Cr@Fe_3_O_4_) on two-week-old zebrafish larvae [93]. A significantly toxic effect (up to 40%) was revealed for all investigated concentrations above 200 *μ*g/mL after 72 h of exposure, with the establishment of a saturation limit of toxicity for doses >600 *μ*g/mL. One possible explanation of this observation is that higher doses did not lead to a higher metabolization rate of the IO NPs. On the other hand, a high concentration-favored increase in agglomeration and precipitation of the IO NPs could cause a reduction of the interaction of the particles with the larvae, ameliorating thus toxicity. In line with this idea, and keeping in mind a potential effect induced by the presence of the conjugated dye, naked NPs showed an effect only at the highest investigated dose of 800 *μ*g/mL. Indeed, administration of Congo red alone reduced the viability starting from concentrations of 50 *μ*g/mL. The revealed highly life stage-dependent toxicity of the investigated IO NPs can be explained by an increased oral uptake by the zebrafish larvae concomitant with an accumulation of the IO NPs in the digestive tract. Indeed, larvae exposed to 100-800 *μ*g/mL of Cr@Fe3O4 revealed a dose-dependent accumulation of particles mainly in the intestine [93]. Indeed, the digestive system is one of the primary sites of iron absorption under physiological conditions and, together with the liver, usually one of the first organs to be affected by IO NP exposure in zebrafish [97, 100]. In this context, the intestinal barrier is known to play a crucial role in the establishment of NP-induced toxicity [85]. According to the size of the implemented IO NPs, the biodistribution can be limited to these organs, as crossing the intestinal barriers can be hampered by the necessity to rely on active transport mechanisms [91].

### 3.2. Effects of IO NPs on Adult Zebrafish

Although much less research concerns the toxicological effect of substances on adult zebrafish, this model system proved important especially in the evaluation of cardiotoxicity [107]. In a study performed by Chemello and colleagues, *γ*-Fe_2_O_3_ IO NPs were exploited as a drug carrier, to facilitate the absorption of antibiotics in adult zebrafish. After 28 days of incubation with oxytetracycline (OTC), tissue accumulation and toxicology markers were assessed for functionalized and naked NPs [109]. In detail, relative quantification of the expression of genes involved in fish stress response (*hnf4a*, *hsp70.1*, *sod1*, *sod2*, and *gsta1*) and growth (*igf1*, *igf2a*, and *mstnb*) was performed in addition to histological analysis of the liver and intestine to elucidate a potential effect. The results showed that uncoated NPs did not induce any alteration in the gene expression pattern concerning the control group. On the contrary, the presence of OCT, alone or on the surface of the IO NPs, led in some cases to a deviation of normal gene expression. In addition, OCT-coated IO NPs showed a reduction of *hsp70* and *sod1* expression concomitant with an increased expression of *gsta1* in the liver of treated fish. These observations are in line with previous studies, revealing a potentially toxic effect of OCT [109]. The authors reported further that the test group receiving the nanocarrier showed a significant increase in drug accumulation, especially in the digestive system. This is in line with previous studies conducted in zebrafish embryos, evidencing the strong ability of IO NPs to be internalized via oral routes and to be then targeted to the intestine tract. They did not reveal any morphological alterations for all analyzed tissues, not for the conjugated nor for the uncoated IO NPs [109], indicating the importance of the status of the organism itself at the moment of administration in the development of teratogenicity. In addition, the fact that no increase in stress response markers was detectable in the presence of the IO NPs is indicative of the absence of any stress response in adults upon IO NP incubation.

A study conducted in 2015 analyzed the effect of static exposure of Fe_2_O_3_ and Fe_3_O_4_ on adult fish in terms of iron accumulation and elimination [95]. For this purpose, adult zebrafish were exposed to the IO NPs at two different concentrations for 28 days and afterward moved to IO NP-free water for 24 days. Interestingly, adult fish exposed to Fe_2_O_3_ NPs displayed a shift in their coloration, probably due to the direct accumulation of IO NPs onto the fish skin, or underlying [95]. In line with what has been described earlier for uncoated IO NPs, both types aggregated in the exposure medium. Since Fe_2_O_3_ precipitated less intensively, their actual exposure to the fish was greater, potentially explaining why the researchers did not observe a shift in the color of the fish treated with Fe_3_O_4_. Independent of the two investigated concentrations (4 and 10 mg/L), a similar amount of internalized iron was revealed in the fish body after 28 days of incubation. However, the amount of body iron did not reach a steady state but declined after reaching a maximum level, indicating that the chronic gut toxicity established as a consequence of the NP exposure induced a reduction of food intake. Given that NPs are taken up by adult zebrafish mostly via ingestion, this led to a decrease in IO NP uptake and thus whole-body iron levels [95].

Another study evaluated the toxicity of IO NPs in the adult zebrafish brain. For this purpose, different doses of cross-linked aminated dextran-coated IO NPs were injected intraperitoneally. Particular emphasis was given on the activity of acetylcholinesterase at different time points after exposure. Only at the highest investigated concentration (200 mg/kg), the enzymatic activity was strongly reduced 24 h posttreatment, concomitant with impaired swimming behavior, indicative of brain toxicity [110]. Although this effect was given only at this time point, no transcriptional regulation seemed to be at its basis. In addition, under the same condition, a significant accumulation of iron in the brain, as well as the increased expression of genes involved in oxidative stress (transcriptional factor AP-1), inflammation (caspase-9), and apoptosis (caspase-8), was observed. Moreover, the oxidative stress markers gclc, *Gpx1a*, *cat*, *gstb1*, and *sod2* were differentially expressed when compared to the control groups. The obtained results are in line with previous findings, where a localized accumulation of IO NPs and thus iron induces oxidative stress, apoptosis, and proinflammatory signaling. In addition, it has been shown several times that mostly high concentrations of IO NPs develop toxicity, underlying the importance to establish carefully the upper limit for each use.

In 2018, Zheng et al. evaluated the effect of 100 mg/L naked and starch-coated Fe_3_O_4_ nanoparticle exposure of 7 days on two different organs, the liver and the gills, known to be a common target for IO NP accumulation [111]. As this concentration of bare IO NPs is known to induce cytotoxic effects in a relatively short exposure time, the authors chose it to discern a possible effect of the coating. To prevent particle aggregation and improve biocompatibility, bare IO NPs are often stabilized or coated with various solvents or chemicals. However, it is widely accepted that the nature of these coatings has an impact on the potential toxicity of IO NPs [111]. Among these, starch coatings are widely implemented in different applications, but especially in the environmental remediation sector. To elucidate this potential effect of the coating on the IO NP-induced toxicity, Zheng and colleagues relied on the transcriptome sequencing (RNA-seq) technique. It was shown that naked NPs accumulated preferentially in the gills where they exerted their toxicity when compared to the coated ones, probably due to higher aggregation and negative surface charge. Indeed, a total of 17 genes involved in immune response, inflammation, oxidative stress, antioxidant response, and endoplasmic reticulum (ER = stress) were differentially expressed upon the treatment. Strikingly, only 3 of these genes were altered in the gills of fish exposed to starch-coated particles, strongly suggesting that the presence of the coated ameliorated notably the toxic effects of the IO NPs on this organ. Bare particles displayed generally higher bioaccumulation in the whole zebrafish when compared to the coated IO NPs. As the gills are one of the first external targets for MO NPs upon exposure, their accumulation in this organ seems logical. However, after surpassing this first entry point, especially the starch-coated IO NPs reach the liver, as their increased biocompatibility renders them more transportable. This is in line with previous findings and further supported by the fact that the liver acts as a reservoir for excess iron and is involved in its excretion [111]. While Zheng and colleagues did not reveal any mortality upon treatment, they noted a noteworthy alteration of gene expression profiles in the investigated organs. More in detail, especially in the case of bare IO NPs, an increase in the gene expression of genes involved in stress response and inflammation was observed. This finding is supported by a wide body of evidence, showing that exposure to IO NPs triggers stress responses and associated toxicity in several in vitro and in vivo model systems [99, 106, 111]. However, the affected sets of genes identified to be altered by IO NPs were only barely overlapping. This difference could be explained by the fact that according to the surface composition of the IO NPs, the interaction with the target organ and thus the activation/inactivation of downstream signaling might be of a different entity. While the presence of the starch coating mitigated the toxic effects at the level of the gills, an increase was observed instead in the liver. This observation is in line with the fact that the coated IO NPs accumulated mainly in the latter one. Indeed, in the liver, both exposures led to an upregulation of genes involved in immune and inflammation responses, concomitant with a downregulation of genes involved in DNA damage and repair. Genes involved in DNA damage/repair and apoptosis (i.e., *tp53*) were differentially expressed especially in the case of starch-coated NPs, indicating the possibility that the nature of the coating is involved in determining mainly this alteration. Interestingly, in the same organ, both forms induced a notable upregulation of the stress gene indicator, cytochrome P450 1 A (*cyp1a*), involved in the antioxidant defense system. In addition, also *tsc22d3*, a marker for an inflammation stress condition, was significantly overexpressed. In line with previous findings, several genes involved in the mitochondrial dysfunction pathway were differentially expressed after exposure to bare particles, further suggesting the production of ROS. Despite the chemophysical properties of the IO NPs, the properties of the main target tissue must be evaluated to assess properly a potentially toxic effect. However, in the study conducted by Zheng et al., both types of IO NPs increased the expression of biomarkers involved in the pathways governing DNA damage, apoptosis, and oxidative stress, in both tissues, indicating that the treated zebrafish were exposed to constant stress.

To assess further the potential ecotoxicity induced by IO NPs accumulating in the environment, another recent study instead investigated the effect of Fe_3_O_4_ MNPs in terms of behavioral and biochemical alterations in zebrafish adults [92]. More in detail, several tests that indicate potential neurotoxicity, such as novel tank, mirror biting, social interaction, shoaling, circadian rhythm, and short-term memory, were performed after 96 h exposure. In particular, the authors assessed two different concentrations, 1 and 10 ppm, with the latter corresponding to the maximal concentration of iron allowed to be present in the Taiwanese industrial waste effluents.

Diverse to what has been described in other studies, Malhotra et al. did not reveal a difference of iron content in ROS levels of the brain, the liver, and the gills of treated and control fish for the investigated concentrations, hinting towards the fact that these kinds of IO NPs are either readily excreted from the adult fish or their uptake from the surrounding environment is reduced [95]. Consequently, it could be hypothesized that potentially associated hazardous effects should be absent for the investigated concentrations. For this purpose, the expression patterns of biomarkers involved in oxidative stress induction and defense were assessed. In line with the absence of IO NP accumulation in this organ, no increase of local ROS levels was observed in the brain of treated fish. In addition, mRNA levels of the enzyme CAT, importantly involved in the antioxidative stress response [106], were significantly increased for the 10 ppm treated groups, indicating a state of stress. It followed that cortisol and catecholamine levels were evaluated, and indeed, an increase of cortisol was revealed again for the highest dose. In addition, hypoxia-inducible factor-1*α* (*HIF-1α*), adenosine-5′-triphosphate (ATP), and creatine kinase (CK) together with markers of DNA damage (ssDNA) were evaluated. Interestingly, only the DNA damage marker revealed a significant upregulation in fish treated with 10 ppm. Exposure to a high dose could thus be associated with the induction of stress response in zebrafish concomitant with elevated brain cortisol levels. Further, the authors showed that an especially high dosage of MNPs could induce several alterations in neurological behavior. For example, a significant correlation was observed in terms of reduced novel tank exploration as well as a reduction in social behavior. Zebrafish are highly social animals, and especially under threat, they tend to swim tightly together. This comportment is known also as shoaling behavior and indicates the anxiety status of the animals. Malhotra and colleagues were able to demonstrate that group formation was tightened in an IO NP dose-dependent manner, indicating increased anxiety in the treated fish. This is in line with the fact that IO NP-exposed fish showed reduced locomotion and exploratory activity for both investigated doses and tended to spend less time at the top of the tank. Furthermore, IO NP exposure led to a considerable reduction in social interaction among the treated fish in comparison to the control group. As no difference was revealed during the mirror biting assay, treated fish did not seem to be more aggressive, although swimming speed was notably increased in the group that has been treated with 10 ppm of IO NPs, underlying a dose-dependent impact on the behavioral patterns. Interestingly, a high concentration of Fe_3_O_4_ NPs affected the circadian rhythm locomotor activity in both light and dark cycles, while 1 ppm induced an alteration only in the light cycle.

These observations were further supported by the fact that exposed fish showed a reduction in the neurotransmitter levels of serotonin, known to be associated with anxiety and depression-like behavior. In addition, the same groups revealed decreased dopamine levels, responsible for stress response, explaining the observed reduction in locomotor activity and aggressiveness. An interesting point in this study is that a high dosage of Fe_3_O_4_ MNPs is correlated to memory deficiency and changes in the levels of the cholinergic neurotransmitter. Indeed, the results revealed that a high dosage of IO NPs had an adverse effect on short-term memory. As reported previously, alteration of AChE is strongly related to neurotoxicity. In line with the behavioral observations made by Malhotra et al., fish treated with both doses of IO NPs showed a significant decrease in AChE when compared to controls. This finding is in line with the study performed by de Oliveira et al. [110], where treatment with high doses of MNP induced a reduction in the enzymatic activity.

## 4. Zinc Oxide Nanoparticles

Zinc oxide is considered a safe material and is approved by the Food and Drug Administration [112]. It is one of the most used metal oxides due to its unique physical and chemical properties, including semiconducting, photo- and sonocatalytic, and piezo- and pyroelectric properties [112, 113]. For all these reasons, zinc oxide nanoparticles have gained scientific interest and present a wide range of applications [114], including cosmetics, optoelectronics [115], ceramics, and pigments; they are implemented as catalysts as well as pain killers and for itch relief.

ZnO nanoparticles present strong antimicrobial properties [5, 6, 116–118]. The antibacterial toxicity of ZnO NPs has been tested against different gram-positive and gram-negative bacteria, such as *Vibrio fischeri*, *Staphylococcus aureus*, *E. coli*, *Salmonella typhimurium*, and *Klebsiella pneumoniae*, showing that higher concentrations of NPs are more toxic [117]. Moreover, in the latest years, ZnO NPs have emerged in the cancer nanomedicine field. As mentioned before, iron oxide nanoparticles are already in clinical use for the hyperthermia treatment of cancer cells. Also, ZnO NPs are implemented in cancer diagnosis and therapeutics due to their unique physicochemical properties and low toxicity impact under certain circumstances [119]. For instance, an immunosensor was developed using ZnO NPs for the early and accurate diagnosis of patients with hepatocellular carcinoma detecting des-carboxy-prothrombin (DCP), which is a highly specific and sensitive biomarker for liver cancer [119, 120]. Taking advantage of their photodynamic and sonodynamic properties, ZnO NPs could be used for exerting remotely cancer cytotoxicity upon an external stimulus, such as light [121], or a mechanical one, like ultrasound [122]. ZnO nanoparticles are also used as targeted and pH-triggered drug delivery systems, as other MO NPs. Indeed, different sizes and shapes of ZnO NPs have been used for this reason, including mesoporous nanospheres and dandelion-like or hexagonal structures [112]. A lot of nanomaterials have been employed in tissue engineering, which is possible due to the easy functionalization methods of their surface with peptides, proteins, and other molecules [112]. As mentioned previously for IO NPs, the biocompatibility of ZnO NPs makes them a good candidate for several biomedical purposes.

Since they can absorb UV radiation, they are commercially used in sunscreens and other products of personal care. In addition to their increasing employment in theragnostic and therapeutics, a lot of questions have been raised on their impact on the aquatic systems [123] and the potentially negative and toxic effects in different organisms. To address the toxicity of these nanoparticles, a lot of studies have been performed on bacteria, plants [124, 125], cells [8, 126], and vertebrates [4, 127].

Understanding the toxicity induced by ZnO NPs turned out to be a quite challenging task for the scientific community. This comes as a large number of parameters contribute to this, such as high experimental condition variability, NP formulation, size, and surface coating [128]. Each of them results in diverse NP physicochemical characteristics that eventually affect the release of Zn^2+^ ions, the reactive oxygen species and photocatalytic ROS production, the pharmacokinetics and biodistribution, and the dynamic interactions with cells [128, 129]. Nevertheless, given the huge potential and advantages of the ZnO NP supersize, researchers focus intensively on approaches allowing to mitigate possible negative aspects while investigating at the same time the optimal working conditions [4, 130]. For this reason, several works have evaluated the potential interactions of ZnO NPs with zebrafish (Table 4).

### 4.1. Effects of ZnO NPs during the Development

Different studies have revealed a dose-dependent toxicity of ZnO NPs in zebrafish during the development [16, 18, 55, 131]. The first work on the toxicological profile of ZnO NPs in zebrafish reported a significant decrease in the survival rate and a delay in the hatching rate, both concentration-dependent, with a value of the LC50 at 96 h of 1.793 mg/L. In addition, larvae presented several abnormalities typical of metal oxide nanoparticle-induced toxicity, including body accusation and pericardial edema [34]. One year later, the same research group demonstrated that the concentration-dependent toxicity of zinc oxide nanoparticles was due to the sedimentation and formation of nanoparticle aggregates (micron-sized) in the experimental plate during the ZnO NP exposure time. Moreover, by using a fluorogenic ROS indicator, an increase in ROS production was detected in treated embryos and larvae. Concomitant with this expression analysis of genes encoding for the oxidant metabolism enzymes, glutathione S-transferase P 2 (*Gstp2)* and NAD(P)H:quinone oxidoreductase (*Nqo1*) revealed a downregulation, hinting thus towards a downfall in the oxidative stress response counteracting normally ROS. Indeed, the impairment of the antioxidant system is known to be associated with the establishment of oxidative stress and injuries. Similar behavior of the previously investigated biological markers was also shown by a subsequent work performed by Bai et al., even if the treated larvae displayed only one malformation, characterized by a severe reduction in the larvae body length [18]. In 2013, Zhao et al. investigated deeply the toxicological profile of ZnO NPs during zebrafish development, focusing also on the DNA damage and oxidative stress. The survival rate did not present any important changes for all the tested groups, while the hatching rate was importantly decreased. In addition, they revealed different morphological malformations, such as tail deformity, spinal curvature, and hyperemia. The study of the antioxidant defense system showed an important elevation in the SOD activity in a ZnO NP concentration-dependent way. In line with this, also the MDA levels were significantly increased in embryos treated with ZnO NPs. CTA activity was instead found to be lower in treated samples in comparison with control ones. Importantly, the level of reactive oxygen species in exposed zebrafish was significantly increased for all the treatments, while the DNA damage level was augmented only at a ZnO NP dose of 100 mg/L [129]. Positive correlations were detected between ROS and DNA damage levels, as well as between ROS and MDA. Moreover, the gene expression analysis of several genes of antioxidant proteins (*Bcl-2*, *Nqo1*, and *Gstp2*) revealed an important downregulation, as previously reported also by Bai et al. [18]. On the contrary, the transcriptome level of uncoupling protein 2 (*Ucp-2*) was importantly upregulated in all the treated groups. These findings underline the fact that ZnO NPs cause adverse effects in zebrafish during the development, leading to an alteration in the expression of genes involved in the oxidation concomitant with oxidative stress [129].

A perturbation in the different toxicological endpoints of zebrafish embryos/larvae treated with ZnO NPs was noted also in other studies [4, 131]. Since Zn^2+^ ions can be released from the ZnO and subsequently transported and uptaken from the embryos, the effects of ZnO NPs and Zn^2+^ were evaluated separately [131]. Zebrafish embryos treated both with ZnO NPs and Zn^2+^ presented a dramatic delay in hatching [131]. The expression analysis by RT-qPCR of specific genes involved in oxidative stress in embryos treated with ZnO NPs and Zn^2+^ showed an upregulation of the cat and Cu/Zn-sod transcripts at 2 dpf, and a downregulation at 3 dpf, respectively, at the highest investigated doses. Instead, eleuthero-embryos showed a downregulation at 5 dpf. On the other hand, the expression of Mt2 was strongly upregulated at 2 dpf and 4 dpf in all the tested doses of both ZnO NPs and Zn^2+^. Moreover, mRNA levels of interleukin-1*β* (*IL-1β*), *TNFα*, and proinflammatory cytokines presented a different expression pattern in eleuthero-embryos in comparison to normal embryos. In eleuthero-embryos treated with Zn^2+^ or ZnO NPs, the *TNF-α* and the *IL-1β* were upregulated, while they were downregulated in treated embryos. Furthermore, an alteration of the jun proto-oncogene (*c-jun*) was detected only in the case of embryos treated with a high concentration of Zn^2+^ and ZnO NPs. In addition, also the antiviral and immune-related gene Myxovirus resistance A (*MxA*) was perturbed in the treatment, both with Zn^2+^ and ZnO NPs. These results indicated that the perturbations induced by Zn^2+^ and ZnO NPs were stronger in the treated embryos in comparison to eleuthero-embryo, indicating that early-stage embryos are more sensitive to nanoparticle exposure [131]. The effects of Zn^2+^ in comparison to ZnO NPs were evaluated also in other studies. Ultrafiltration and ICP-OES allow to calculate the dissolved Zn ions [16]. The concentration of Zn ions keeps increasing over time and is transduced in an increase of pH [16, 18]. The presence of released Zn^2+^ ions derived from the ZnO NPs could explain the low hatching rates [4]. However, this is not yet clarified and there are different contradictory studies, some of which support the same conclusion, while others claim that ions contribute only partially to the low hatching rate. Chen et al. compared the adverse effects of ZnO NPs in zebrafish during the development in comparison to Zn ions. Both treatments induced a hatching delay that was more severe in the groups of embryos treated with ZnO NPs, rather than in those exposed to Zn ions alone. However, as Zn ions did cause a delay in hatching, it can be concluded that the toxicity on hatching is probably caused by a combination of different factors. Indeed, the presence of released Zn^2+^ ions contributes to this. The induced ROS generation and, consequently, the oxidative stress could be another reason [132]. To understand better the cause that leads to the hatching delay, Chen et al. coexposed the embryos to ZnO NPs and NAC or buthionine BSO. In the groups cotreated with ZnO NPs and NAC, no significant difference was observed. However, treatment with BSO further increased the rates of delay in hatching. Moreover, when GSH was no longer synthetized due to the presence of BSO, further hatching delay was observed, suggesting that oxidative stress could be related to the hatching delay along with Zn^2+^ ion release [132].

In the same year, another study evaluated the effects of zinc oxide nanoparticles with different shapes, including submicron particles, nanosticks, and nanospheres [133] and Zn(NO_3_)_2_. The LC50 values for Zn(NO_3_)_2_, ZnO SMPs, nanosticks, and nanospheres at 120 hpf were 7.9 (7.1–8.8) mg Zn/L, 10.0 (8.9–11.1) mg Zn/L, 7.1 (6.8–7.5) mg Zn/L, and 11.9 (10.3–13.7) mg Zn/L, respectively, reporting higher toxicity of Zn ions in comparison to the differently shaped NPs. The hatching rate showed a dose-dependent decrease in the embryos treated with all the different kinds of nanoparticles and sulfate, with the strongest delay in samples exposed to nanosticks. Besides this, the swimming activity displayed a dose-dependent decrease. The ZnO nanosticks were found to be more toxic in comparison to nanoparticles with other shapes [133].

**Figure 3:**
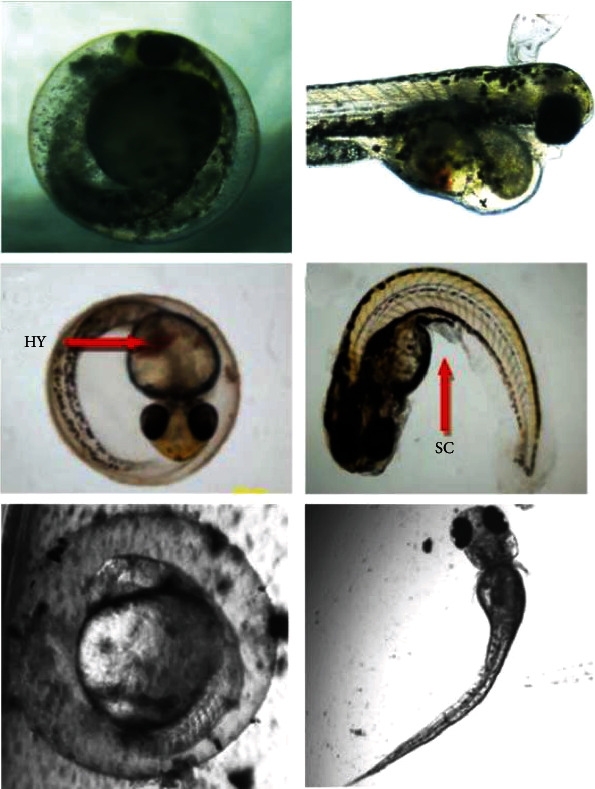
Malformations induced by ZnO NPs in zebrafish during the development. Reproduced with permissions from [17, 135, 137].

To determine the contribution of Zn^2+^ ions in the toxicity of ZnO NPs, ZnCl_2_ or ZnSO_4_ exposure is used to compare experimentally the toxic effects [16, 17]. Choi et al. performed toxicity experiments exposing the embryos to ZnO NPs and ZnSO_4_ to compare eventual effects on zebrafish development. As it is also shown in other studies, exposure to nanoparticles leads to a higher rate of mortality than exposure to only ZnSO_4_. The LC25 values for ZnO NPs were 2.64 mg/L and 7.75 mg/L for ZnSO_4_ [17]. However, embryos showed significant embryonic malformations after both treatments, including tail malformation, pericardial edema, and yolk sac edema, indicating an adverse impact given by the presence of nanoparticles and Zn ions (Figure 3) [17]. In particular, embryos exposed to all the tested doses of ZnO NPs presented a yolk sac edema. After an extended analysis of differentially expressed genes (DEGs) in larvae zebrafish, it was shown that exposure to ZnO NPs and ZnSO_4_ affects different molecular mechanisms and subsequently causes distinct toxic effects. In particular, the treatment with ZnO NPs altered genes involved in the immune system inflammation. Indeed, the expression of *ogfrl2* (opioid growth factor receptor-like 2 [ogfrl2]) and Intelectin (2*intl*2) was upregulated after treatment with ZnO NPs, while cytochrome b5 domain containing 1 (*cyb5d1*) was upregulated. Together with *cyb5d1*, *Ogfr12* and *intl2* play an important role in developmental and transcriptional regulation, and both genes were upregulated after treatment with Zn nanoparticles [17].

Wehmas et al. instead found that Zn ions caused the same mortality rate and affected zebrafish development in the same way as ZnO NPs. This result suggests that the toxicity is mainly due to the presence of dissolved zinc ions [16]. But again, also in this case, not all the reported studies arrive at the same conclusion. Indeed, Xiong et al. observed that all the zebrafish embryos died at a concentration of 30 mg/L ZnO NPs or their bulk counterpart after 96 h of exposure [9]. In addition, a high toxicity rate was observed in the group of embryos treated with Zn^2+^ ions. The LC50 values were 4.92 mg/L, 3.31 mg/L, and 8.062 mg/L for ZnO NPs, ZnO bulk, and Zn^2+^ ions, respectively. As a result, the released Zn2^+^ ions could not be the main cause of toxicity, but it is rather the combination of nanoparticles with ZnO [9], as in the case of other metallic oxide NPs.

As for other metal oxide nanoparticles, the influence of coating and size on the toxicity of zinc oxide nanoparticles was evaluated [116]. To this end, zebrafish embryos were treated with 17 different types of ZnO NPs, and they were investigated in terms of 19 different toxicological endpoints, including morphological and behavioral tests. The biological parameters that resulted to be more affected in all the tested nanoparticles were the mortality/survival rate. In particular, all tested and differentially coated nanoparticles induced significant mortality at 24 hpf, while the bare nanoparticles did not lead to important alterations until 5 dpf. These findings indicate that the surface coating, as in the case of other families of nanoparticles, is a key factor influencing the adverse effects in biological systems [134]. Next, the effects of ZnO NPs functionalized with polymeric surface-modifying agents including polyvinyl alcohol (PVA), polyethylene glycol (PEG), and polyvinylpyrrolidone (PVP) were evaluated in zebrafish during the development [130]. The treatment with the bare or the capped nanoparticles caused morphological defects, such as yolk sac edema, notochord bending, and egg coagulation. Indeed, the rates of toxicity reported after treatment with 10 mg/L were 38.67%, 28.49%, 95.46%, and 89.32% for PEG-, PVA-, and PVP-capped and bare ZnO NPs, respectively [130]. It was demonstrated that the NP toxicity is a combination of the toxicity caused by dissolved Zn ions and aggregation of nanoparticles on the eggs, shown by the increased toxicity caused by capped or bare ZnO NPs in comparison to bulk ZnO (bulk ZnO < ZnO − PVA < ZnO − PEG < ZnO NPs < ZnO − PVP) [130].

The effects of size and surface charge were deeply investigated by Verma et al. [135]. Here, they evaluated a potential impact on the zebrafish development of different ZnO nanoparticles produced by the HEBM technique relying on a variety of milling times [55, 56]. They showed that decreasing the size and the charge influenced proportionally both the survival and hatching rates of treated embryos. This observation was confirmed also in the case of the heartbeat rate and incidence of malformations (Figure 3). In addition, ZnO NPs induced an increase in ROS production in zebrafish larvae and embryos, as already observed in previous works [134, 136].

Several studies have also reported that treatment with ZnO NPs affects the expression of different genes which play a crucial role in oxidative stress and inflammation [116]. The expression and activity of CAT and SOD and the levels of MDA were evaluated in zebrafish embryos exposed to different concentrations of ZnO NPs [116, 136]. Embryos treated with ZnO NPs in a concentration range of 30-120 mg/L showed an increase in lipid peroxidation and in SOD activity and revealed perturbations of genes involved in the antioxidant defense mechanism. In particular, an upregulation was found in the expression of *ppaα* and *sod1*, while *cat* was downregulated. To understand if such an increase in SOD activity was caused by the released Zn^2+^ ions, Zhao et al. treated zebrafish embryos with dissolved ions. They concluded that dissolved Zn^2+^ ions in concentrations below 60 mg/L do not lead to increased SOD activity. This means that the upregulation of SOD cannot be caused only by the release of Zn^2+^ ions from the nanoparticles, but other factors contribute to it [116]. Furthermore, the production of ROS, the basis of oxidative stress, was confirmed by an altered expression of antiapoptotic genes (*Bcl-2,* B-cell lymphoma 2) and proapoptotic (*bax, puma*, and *apaf-1*) genes. In addition, the transcription of the *p53* gene was upregulated causing an augmentation of p53 and cytochrome C protein levels. To determine the effect of ZnO NPs on apoptosis, also the expression levels of genes related to apoptosis (antiapoptotic and proapoptotic genes) were evaluated. Augmenting the concentration of nanoparticles, a higher apoptotic ratio was observed in a dose-dependent manner (10-120 mg/L), corresponding to a significant increase in the activity of caspase-3 and caspase-9. As the expression of ROS is involved in the mitochondrial pathway responsible for the induction of apoptosis, it is plausible that its accumulation leads to a concomitant increase in MDA levels. In this framework, Du et al. treated zebrafish embryos with different doses of ZnO NPs and measured the activity of antioxidant enzymes at 96 hpf [136]. In agreement with the previous results, the SOD activity was significantly increased even in embryos treated with the lowest concentration of nanoparticles, as well as the activity of glutathione peroxidase. In contrast, the CAT activity was decreased in a dose-dependent manner. However, despite this reduction, the expression of *cat* was not different from the control. The levels of intracellular ROS were analyzed by the cell-permeable dye DCFH-DA. ROS levels were highly increased in a dose-dependent manner in the groups of zebrafish exposed to ZnO NPs [136]. Moreover, the cellular content was evaluated. As for the ROS, the MDA levels were significantly increased after treatment with 25 and 50 mg/L of ZnO. While the expression of BCL2-associated X apoptosis regulator (*Bax*) was significantly upregulated, *Bcl-2* resulted to be downregulated after treatment with 50 mg/L for both conditions. These findings are in agreement with the study conducted by Zhao et al. [116]. The only difference was related to the caspase activity. In fact, Du et al. did not find any changes in the activities of the caspase-3 and caspase-9 after exposure with ZnO NPs for 96 h [136]. However, the expression of caspase-3 was upregulated in the groups treated with 25 and 50 mg/L of ZnO NPs, with caspase-9 resulting to be upregulated in the group treated with 50 mg/L. Both studies, as well as others, conclude that apoptotic cell death is mediated by oxidative stress [116, 136].

### 4.2. Effects of ZnO NPs on Adults

The first work performed on ZnO NPs and adult zebrafish has deeply investigated the adverse and oxidative effects of this class of nanoparticles in comparison to the titanium nanoparticles and their bulk counterparts [9]. To this end, adult zebrafish were exposed to different concentrations of NPs and bulk materials for 96 h. It was shown that the toxicity of ZnO NPs and bulk ZnO was dose-dependent. Treatment with 30 mg/mL of ZnO NPs and bulk ZnO led to a 100% of mortality. The values of LC50 at 96 hpf were found to be 4.92 mg/L and 3.31 mg/L, for the ZnO NPs and bulk ZnO, respectively. Zebrafish treated under light or dark conditions with the highest dose of ZnO NPs and bulk ZnO presented a temporary increase in liver activity of SOD in the gut, compared to the controls. Interestingly, in the groups of zebrafish treated with bulk ZnO, the SOD activity was less than the control in both tissues [9]. The CAT activity instead was reduced in the liver, whereas in the gut and gill (but only slightly), it was shown to be increased. Glutathione was decreased in the liver tissue after 96 h of exposure, probably due to ROS that neutralized it. However, in the gut tissue, an increase in GSH content was detected after exposure to the ZnO NPs, but not to bulk ZnO. Moreover, the MDA levels were two or three times higher in the case of the liver, while they were similar to the control in the case of gut and gills. As reported before, ZnO NPs can impair the maturation of gills and cause developmental defects [16, 130]. In addition, histological analysis performed by using transmission electron microscopy (TEM) revealed injuries in the gill tissue after treatment with the lowest doses tested of ZnO NPs and bulk ZnO. Here, cells displayed shrinkage, loss of the cytoplasm, and abnormalities in the nuclei shapes. It is worth mentioning that as for TiO_2_ NPs, ZnO NPs in suspension can generate OH ions after illumination with fluorescent light. Interestingly, the bulk ZnO or ZnO did not generate any OH in a dark environment. Moreover, at the concentration of 5 mg/L, the amount of produced OH^−^ was quite low. Due to this low concentration of·OH^−^, and the lack of significantly increased levels of oxidative indicators in the gills, the damage of the gill cells could not be caused by the induction of ROS and oxidative stress. Hence, Xiong et al. stated that a different mechanism had to be at the basis of the gill tissue damage. Since zebrafish ingested the NPs mainly with the diet, the liver was the tissue mostly exposed to the nanoparticles and consequently mainly affected [9].

As mentioned in the case of zebrafish embryos/larvae, different studies have been focused their attention on the impact of surface modifications on the toxicity of ZnO NPs. Kizhakkumpat et al. investigated these factors not only in embryos and larvae but also in adult zebrafish. Adult fish were treated with ZnO-PEG, ZnO-PVA, and ZnO-PVP NPs. In embryos, the LC50 of bulk ZnO, ZnO-PEG, ZnO-PVA, ZnO-PVP, and ZnO NPs were found to be 520.9, 17.21, 131, 0.6823, and 0.7579 mg/L, respectively. However, the LC50 values in adults were 3239, 6.44, 9.40, 3.77, and 20.72 mg/L. In addition, capped ZnO nanoparticles were taken up by the embryos at higher rates than bare ZnO NPs, resulting in increased toxicity in later life stages. Moreover, as ZnO-PVP NPs showed the highest uptake level, adult zebrafish had a lower survival rate when exposed to this form of nanoparticles [130]. Furthermore, adult zebrafish treated with these different kinds of nanoparticles showed different morphological alterations. In particular, the histopathological study revealed severe damage in the gill tissue. More specifically, secondary lamellar structure alterations, necrosis, desquamation, acute cellular swelling, aneurysm, and lamellar disorganization were observed [130]. This specific effect was already noticed in the previous studies elucidating the toxicity of ZnO NPs in zebrafish embryos. Larvae treated with ZnO NPs and Zn^2+^ presented specifically tissue ulcerations and gill primordia. These findings are in agreement with work of Kizhakkumpat et al. and clearly indicate that ZnO NPs cause diverse toxic effects relevant to the stages of zebrafish life, with later life stages being more sensitive than the embryonic ones [16].

## 5. Conclusions

Current research suggests that exposure to metallic nanoparticles, especially when administered in high concentrations, causes adverse effects in zebrafish. Although TiO_2_ NPs, IO NPs, and ZnO NPs are widely approved and considered nontoxic, they can indeed present some harmful properties. All the three nanoparticles types have in common that the contribution given by their size (core and hydrodinamic), coating, as well as by the experimental conditions themselves, need to be considered in the establishment of their toxicity. Importantly, several studies showed that the accumulation of highly concentrated metallic oxide nanoparticles, concomitant with the release of their appropriate ions, is at the basis of the observed nanotoxicity. Especially in combination with longer exposure times, this seems to play a crucial role in the induction of ROS and the activation of related inflammatory and/or immunogenetic mechanisms for all classes of investigated NPs. Particularly the exposure to the highly investigated TiO_2_ NPs has been revealed to affect these pathways. It has been shown that alteration of *sod1* activity with consequent perturbation of *tp53* impacts lipid homeostasis while promoting genotoxicity and apoptosis. Given their particular optical properties, these effects can even deteriorate under illumination, demanding for an accurate evaluation of their potential toxicokinetics prior to their implementation. Similarly, ZnO NPs can cause an increase in reactive oxygen species in response to fluorescent light. Furthermore, the ZnO NP-induced steep increase of ROS stimulates the apoptotic pathways regulated by caspases and mitochondria (*Gstp2, Nqo1, Bcl-2*, *caspase*-*3*, and *caspase*-9) causing extensive cellular dysfunction even at lower concentrations. Regarding IO NPs, which are associated with oxidative stress and the induction of redox-sensitive signal transduction pathways (AP), nanoparticle size and coating seem to be the factors mostly contributing to the observed cellular dysfunction. As iron ions are important components of many biochemical reactions, its concentration must be tightly controlled although when administered as IO NPs. Despite the increasing implementations of MO NPs, and the constant development of new variants, the obtained results regarding nanotoxicity are often contradictory. Taken as a whole, caution must be thus advised in the usage of all the indicated nanoparticles. This is even more important as MO NPs are widely used in several daily life applications, leading inevitable to environmental and human exposure. It is clear that further research is needed to fully unravel the mechanisms underlying nanotoxicity in organisms upon MO NP exposure to mitigate as much as possible potentially occurring adverse effects. In addition, proper evaluation of their ecotoxicological profile demands strongly for the standardization of the experimental conditions.

## Figures and Tables

**Table 1:** Properties and applications of the most used metal oxide nanoparticles.

Metal oxide nanoparticles	Physical, chemical properties	Potential applications in medicine (tested *in vitro*/*in vivo*)	Biomedical and life science applications (in use and commercial products)	References
Aluminium oxide (Al_2_O_3_)	Catalyst, high thermal and mechanical stability, high corrosion resistance, and high melting point.	Drug delivery.	—	[18]
Copper oxide (CuO)	Catalyst, high-temperature superconductors.	Anticancer treatment.	Antimicrobial coating agents.	[2, 11]
Iron oxide (*α*-Fe_2_O_3_, *γ*-Fe_2_O_3_, and Fe_3_O_4_)	Superparamagnetic and magnetic hyperthermia properties, catalyst.	Antibacterial agent, drug delivery, anticancer treatment (photothermal therapy, chemotherapy, and magnetic hyperthermia therapy), and theragnostic (near-infrared imaging, positron emission tomography, single-photon emission computed tomography, and ultrasound imaging).	Iron-deficient anemia treatment (Venofer®, Feraheme®, and Rienso®).	[2, 19]
			Solid tumor treatment (NanoTherm®).	
			Magnetic resonance imaging (in liver: Feridex I.V.®, Endorem®, and Resovist®; in gastrointestinal: Gastromark™ and Lumirem®; and in blood pooling: Supravist®).	
Magnesium oxide (MgO)	High ionic character, catalyst, and semiconductor.	Antibacterial agent, anticancer treatment (hyperthermia therapy), and tissue engineering.	Antimicrobial agents (in food industry).	[2, 18]
Nickel oxide (NiO)	Catalyst, magnetic properties, and high electrochemical stability.	Anticancer treatment (cytotoxic properties).	—	[11]
Silica dioxide (SiO_2_)	Low density.	Antibacterial agent, drug and gene delivery, anticancer treatment, and biosensor.	Additive in drugs, cosmetics.	[2, 11, 13, 20]
Titanium oxide (TiO_2_)	Semiconductor, photocatalyst, and high chemical stability.	Anticancer treatment (photodynamic, photothermal, sonodynamic therapy, chemodynamic therapy, and radiotherapy), theragnostic (bioimaging), drug delivery, and tissue engineering.	UV-A, UV-B radiation filter (in sunscreens, cosmetics).	[2, 8, 11, 19, 20]
			Antimicrobial agents (in food packaging, biomedical devices, and dentistry & orthopedic implants).	
Zinc oxide (ZnO)	Semiconductor, photocatalyst, high chemical stability, large exciton binding energy, and high isoelectric point.	Anticancer treatment (photodynamic, photothermal, and sonodynamic therapy), theragnostic (bioimaging), drug delivery, and tissue engineering.	UV-A, UV-B radiation filter (in sunscreens, cosmetics).	[2, 4, 9, 11]
			Antimicrobial agents (in toothpaste, dentistry implants, food packaging, and as food additive).	

**Table 2:** Impact of TiO_2_ NPs on zebrafish.

Stage	NPs diameter	Treatment duration	Tested concentrations	General toxicity response	Specific ROS responses	Reference
Embryos	30 nm	48 h	Up to 10 mg/L	No toxic effects.	—	[35]
Embryos	≤20 nm	96 h	1, 10, 50, 100, and 500 mg/L	No significant differences in survival, hatching, and malformation rates.	—	[34]
Embryos	27.7 nm	120 hpf	0.1, 0.5, 1, 5, and 10 mg/L	No significant differences in survival and hatching rates; reduction in average swimming speed at 120 hpf at low concentration; and no changes after the coexposure with NAC or BSO.	—	[37]
Embryos	86 and 409 nm	96 hpf	170 ng/mL+40 *μ*g/mL hydroxylated fullerenes/C60(OH) 24)	—	Downregulation of genes associated with circadian rhythm, transport and vesicular trafficking, and immune response.	[48]
Embryos	23.3 nm	120 hpf	1, 10, 100, 500, and 1000 *μ*g/mL	LC50 = 300 *μ*g/mL with no light; LC50<1000 *μ*g/mL with light; at 8 days all illuminated larvae died at 100 *μ*g/mL; and different malformations (head, tail, yolk, and heart).	ROS generation in presence of light; oxidative stress response in transgenic line; and DNA damage with TiO_2_ NPs ≤1000 *μ*g/mL under illumination.	[45]
Embryos	5, 10, and 21 nm	Over 23 days	0.01-10 000 ng/mL	Significant mortality rate (speed up with light); reduction in size, deformations of craniofacial structures and absence or abnormal organization in the pigmentation; and swim bladder with a single lobe.	Significant oxidative stress and intracellular damages.	[46]
Embryos	4, 10, 30, 50, and 134 nm	48 h	50, 500, 5000, 25000, and 50000 *μ*g/L	No effects on zebrafish with 4 and 30 nm NPs; low impact on the mortality rate at 5000 and 250000 *μ*g/L with 10 nm and 134 nm NPs.	No necrotic cells or a low amount of them for all the different size and doses tested; normal expression of Mt2.	[59]
Embryos	21 nm	72 hpf	1 mg/L	No effects on mortality rate; no significant incidence of malformations; expression of *atho7* in the retina similar to controls; all the components of the retina well differentiated; and no effects on the neurogenesis.	—	[51]
Embryos	7.04 nm	7 dpf	0.1 mg/L+BDE (0.08 and 0.38 mg/L)	Similar survival and hatching rates of the sample treated with BDE or BDE plus NPs; important increase in T4 values in cotreated samples; no difference in T3; important upregulation in the expression of the *tg*, *tshβ*, and *dio2* genes; downregulations of *α1-tubulin* and *mbd* genes; perturbations in the expression of the *mbd* protein; and reduction in the swimming speed.	—	[50]
Embryos	≤25 nm	96 hpf	10 and 50 mg/L +5 and 10 mg/L of BPA	TiO_2_ NPs: normal survival rate; no important malformations; and decreased hatching rate at the highest dose tested TiO_2_ NPs+BPA: significant decrease dose-dependent of survival rate, different abnormalities (spine deformation, weak pigmentation, and pericardial edema).	—	[52]
Embryos	NM-103/104: 20 nm; P25: 21 nm; and micro-TiO_2_: 200 nm	8 dpf	0.01, 0.1, and 1 mg/mL	No effects on survival, hatching, or deformities rates; decrease in the length of larvae at one dose of microsized TiO_2._.	Decrease in SOD activity; perturbation in GSH levels; and highest levels of ROS in embryos treated with P25 NPs.	[54]
Embryos	25 nm	6 dpf	0.1 mg/L+PCP (3, 10, and 30 *μ*g/L)	Similar survival and hatching rates in samples treated with PCP and PCP plus nanoparticles; incidence of malformations higher in coexposed larvae.	Alterations in GSH content, SOD activity and MDA in sample treated with only NPs; decrease in the SOD activity and GSH content and important levels of MDA and ROS in cotreated samples; and an important upregulation *sod1* and *nrf2* in cotreated samples.	[49]
Embryos	6, 12, and 15 nm	120 hpf	0–1000 *μ*g/mL	LC50 6 nm: 23 *μ*g/mL; LC50 for 12 nm: 610 *μ*g/mL LC50 for 15 nm: not detectable; several phenotypic abnormalities (opaque yolk, axial curvatures, craniofacial defects, yolk sac, and pericardial edema).	High levels of hydroxyl radical (˙OH) and ROS; higher values for 6 nm NPs in comparison to 12 and 15 nm NPs.	[47]
Embryos	Anatase, TA <25 nm; anatase/rutile mixture, TM, form, 25 nm	96 h	1, 10, and 100 mg/L	5% of mortality only after 96 hpf in the group treated with 100 mg/L of TA under UV light; lower hatching rate in zebrafish treated with TA and under UV illuminations; egg coagulation and perturbations in equilibrium in zebrafish treated with TM; and significant decrease of survival and hatching rates under UV light.	Under UV illumination decrease in the enzymatic activity of AP, GST, and CAT; state of oxidative stress.	[68]
Embryos	7.02 nm	6 dpf	0.1 mg/L+Pb (0, 5, 10, 20, and 30 *μ*g/mL)	Effects on organogenesis in coexposed larvae; decrease in T3 and T4 levels in zebrafish treated with 30 *μ*g/mL of Pb alone or to all the doses of Pb plus TiO_2_ NPs; downregulation of *tg* and TTR *shha*, *gfap*, *α*-tubulin, and *mbp* genes; upregulation in *tsβ* gene; and significant decreased in the swimming speed.	—	[61]
Embryos	50-70 nm	96 hpf	0.1, 1, and 10 *μ*g mL	No alteration in survival rate; decrease in hatching rate; significant incidence of abnormalities (tail flexure and pericardial edema); decrease in total distance of swimming; and TiO_2_ NPs able to cross the BBB, localized in the larvae brain.	High ROS production with consequent oxidative stress; high apoptosis in the hypothalamus region; upregulations of the genes *α*-*syn*, *parkin*, *uchl1*, and *pink1*; and decrease in the dopaminergic neurons.	[60]
Embryos	Bulk TiO_2:_ ∼110 nm; 5 h TiO_2_ NPs: 85 nm; 10 h TiO_2_ NPs: 62 nm; 15 h TiO_2_ NPs: 46	96 h	10-250 *μ*g/mL	Significant decreased or increased, respectively, in a dose-dependent manner of survival rates and hatching rates; strongest effect for embryos/larvae treated with TiO_2_ NPs milled for the longer time (15 h).	ROS quenching; steatosis, lipid accumulation in dose-dependent manner in different areas of the animal (tail, head, and notochord); high number of apoptotic cells in tail and head; perturbation of *sod1* protein activity; and perturbation of protein *tp53*.	[56]
Adults	<150 nm	5 days	1, 2, and 4 mg/L	Structural changes and degeneration of the follicles.	Several vacuolizations in the cytoplasm; evident forms of paraptosis; mitochondrial vesiculation and chromatin condensation; and swelling and mitotic catastrophe.	[69]
Embryos	5–25 nm	72 hpf	500 and 1000 mg/L	No changes in the survival rate for all the treated samples.	Perturbation of *SOD2* mRNA level both under illumination and in dark condition; normal level of *Pxmp2*; and significant difference in *IF1* mRNA level under illumination.	[65]
Embryos	20 and 30 nm	96 h	1, 10, 50, and 100 *μ*g/mL+10 *μ*g/mL	TiO_2_ NPs: survival rate of 85%: TiO_2_ NPs+HA: 95%. HA decrease harmful effects of TiO_2_ NPs.	—	[53]
Embryos	40 nm	96 h	10, 25, 50, 100, 250, and 500 *μ*g/L	LC50 = 90 *μ*g/mL; enhancement of hatching rate of embryos; and some abnormalities (both body and organs).	Lower ROS production for the TiO_2_ NPs produced by HEBM method, compared to the bulk one.	[56]
Embryos	1-3 nm		10, 100, and 1000 mg/L	100% mortality at the highest concentrations; delay in hatching rate at the middle and highest doses tested; several malformations (aneurysm and pericardial edema) in embryos injected with TiO_2_ USNPs; any perturbations or vascular toxicity in the ones injected in the circulatory systems at 48 hpf; length reduction of the ISVs in eggs treated by soaking or injection with 100 mg/L of TiO_2_ USNPs; and perturbation in *Myo1c* expression.	—	[58]
Embryos	21 nm	34, 58, 82, 106, and 130 h	0.01, 10, and 1000 mg/mL	73% of embryos exposed to highest dose hatched prematurely between 34 and 58 hours post exposure.	—	[57]
Embryos	5 nm	2 days	100 *μ*g/L) TiO_2_ NPs+Pb (0, 10, 20, and 40 *μ*g/L); a subsequent depuration (144 h)	Survival and hatching rates up to 85% for all the investigated cases; significant perturbation in these biological parameters observed only in at 40 *μ*g/L Pb plus TiO_2_ NPs; and reduction in the larvae swimming speed.	—	[62]
Embryos	Micro-TiO_2_ 1–2 *μ*m Nano-TiO_2_ 21 nm	6 dpf	0.01, 0.1, and 1.0 mg/L nano-TiO_2_ and 1.0 mg/L micro-TiO_2_	No effects on survival and hatching rates; body weight and length of larvae decreased as well as rotation times and the swimming speed; perturbation in the neurogenesis and in the motor neuron axon length; and perturbation in the expression of genes *α1-tubulin*, *mbp*, and *gap43*.	—	[63]
Adults	20.5	48 h	1000 *μ*g/L	No significant alterations in gill histopathology; important changes in the expression of 171 genes (111 genes downregulated and 60 upregulated).	—	[36]
Adults	21 nm	14 days	0.1 or 1.0 mg/L	No behavioral abnormalities and no mortality; changes in the number of white blood cells at the last day of exposure (14) for all the tested doses of TiO_2_ NPs.	Normal Na^+^K^+^-ATPase activities in the liver, gill, and brain; values of GSH in the liver, gill, and brain higher in comparison to controls; histology of all these tissues normal; and absence of intracellular oxidative damage.	[67]
Adults	9.7 nm	90 days	100 *μ*g/L+0, 2 and 20 *μ*g/L BPA	Change in the intestinal microbial community after cotreatment of TiO_2_ NPs and BPA.	Oxidative stress and inflammation dose-dependent and sex-dependent; oxidative responses due to the cotreatment linked to a different amount of *Lawsonia* and *Hyphomicrobium.*	[72]
Adults	<150 nm	5 days	1, 2, and 4 mg/L	Swelling and loss of cristae and degenerated mitochondria in spermatocytes and Sertoli cells; high amount of necrotic cells; and damages in the testicular morphology and negative impact on the fertility.	—	[70]
Adults	23.8 nm	5, 7, 14, 21, and 28 days	1 and 10 *μ*g/L	—	Significant percentage of DNA fragmentation with maximum injuries after 14 days; significant number of apoptotic cells; and important decrease of genome stability (GTS%) at 14 days, and then recovered in part at 28 days.	[71]
Adults	240–360 nm	91 days	0.1, 1.0 mg/L	After 9 weeks, decreased number of embryos; increase in mortality rate at 2 dpf of embryos produced by the exposed female; perturbation in the follicular stages, with a block in the development; and important alteration of genes involved in the development of oocytes.	—	[66]
Embryos and adults	25 nm	Embryos: 96 hpf Adults: 7 days	Embryos: 10, 50, and 100 mg/L Adults: 10, 50, and 100 mg/L	—	Embryos: no effects on hatching rate, no sign of deformity. Adults: significant decrease of activities of GSTs, CAT, and SOD in the gills and liver; oxidative stress condition.	[64]
Adults	21 nm	21 days,	5 and 40 mg/L	Increase of both bacteria (gut) in the water and animal motility; *Actinobacteria*, *Bacteroidetes*, and *Proteobacteria* main component of the flora of the gut.	—	[73]

Abbreviations: AP: acid phosphatase; *atho7*: atonal homolog; BDE: polybrominated diphenyl ethers; BBB: blood-brain barrier; BPA: bisphenol A; BSO: buthionine sulfoximine; CAT: catalase; *dio2*: iodothyronine deiodinase 2; *gap-43*: growth-associated protein 43; *gfap*: glial fibrillary acidic protein; GSH: glutathione; GST: glutathione S-transferase; HEBM: high-energy ball milling; *HIF1*: hypoxia-inducible factor 1; HA: humic acid; hpf: hours post fertilization; ISVs: growing intersegmental vessels; LC50: 50% of lethal concentration; MDA: malondialdehyde; *mbd*: methyl-CpG-binding domain; Mt2: metalloprotein 2; *Myo1c*: Myosin IC; NAC: N-acetylcysteine (NAC); *Nrf2*: nuclear factor erythroid 2-related factor 2; PCP: pentachlorophenol; *Pxmp2*: peroxisomal membrane protein 2; ROS: reactive oxygen species; *shha*: hedgehog protein A precursor; SOD: superoxide dismutase; TA: anatase; *tg*: thyroglobulin; T3: triiodothyroxine; T4: thyroxine; TM: anatase/rutile mixture; *tp53*: tumor protein 53; *tshβ*: thyroid-stimulating hormone *β*; *uchl1m*: ubiquitin C-terminal hydrolase L1; USNPs: ultrasmall nanoparticles.

**Table 3:** Impact of IO NPs on zebrafish.

Stage	NP diameter	Treatment time	Tested concentrations	General toxicity response	Specific ROS responses	Reference
Embryos	22 nm	144 h	0.3; 0.6; 1.25; 2.5; 5; and 10 mg/L	High mortality rate; cardiotoxicity (reduction of heart beat rate); and morphological alterations.	—	[3]
Embryos	6-12 nm	120 hpf	SP IONs, S PION-DX, SP ION-CS, SP ION-T, SPI ON-T-PEG, SP ION@SiO_2_: 0.125 mM, 0.5 mM, 2.0 mM, and 8.0 mM	SP ION-CS: reduced survival rate, SPI ON-CS, and SP ION@SiO_2_ delay in hatching rate; SP ION-DX, SP ION-T-PEG and SP ION-T: slightly premature hatching; SP ION-CS and SPI ON@SiO_2_: reduction in locomotor activity; and SP ION-CS, SP ION-T-PEG SP ION@SiO_2_ reduction in escape behavior.	—	[15]
Embryos		168 hpf	0.1, 0.5, 1, 5, 10, 50, and 100 mg/L	Mortality concentration and exposure time dependent; LC50 = 53.35 mg/L; delay in hatching rate, LC50 = 36.06 mg/L; and different malformations (pericardial edema, tissue ulceration, and body arcuation).	—	]76]
Embryos	40 nm	96 h	Fe_3_O_4_ NPs: 100-800 *μ*g/mL bare Cr@Fe_3_O_4_: 5, 150, 300, and 600 mg/mL	Fe_3_O_4_ NPs: dose- and time-dependent delay in hatching rate; slight decrease in embryo viability; Cr@Fe_3_O_4_: NPs high mortality in 2-week-old larvae; dose-dependent accumulation in digestive tract.	—	[93]
Embryos	100-250 nm	168 hpf	1, 5, 10, 50, and 100 mg/L	LC50 = 10 mg/L; delay in the hatching rate.	—	[97]
Embryos	22-45 nm	96 hpf	10, 20, 40, 60, 80, 110, 120, and 140 ppm	LC50 =60.17 ppm; delay in hatching rate; reduction in heart beat rate; and increased teratogenicity.	Dose-dependent decrease of Na+K^+^-ATPase activity; dose-dependent increase of AChE; increased levels of lipid peroxidation ROS, PC, and NO; increase of apoptotic bodies; and decrease of antioxidant enzymes, CAT, SOD, and Gpx.	[98]
Embryos/adults	15 nm	Embryos: 96 hpf Adults: 2 weeks	Embryos: 1, 10, 100, and 1000 ppm Adults: 1, 10 ppm	Embryos: no adverse effect observed Adults: reduced locomotor and exploration activity, increased anxiety, reduced social interaction, tightened shoaling behavior; dysregulation of circadian rhythm locomotor activity, reduction of short-term memory retention, and reduction of serotonin and dopamine.	Increased CAT, cortisol level in the brain; reduction of AChE activity.	[92]
Adults	21 nm	7 days	100 mg/L	Bare IO NPs accumulate mainly in the gills, coated IO NPs in the liver.	Altered expression of genes involved in inflammation, immune response, oxidative stress, antioxidant response, and mitochondria in the gills of Fe_3_O_4_-treated fish. Upregulation in the liver of genes involved in immune and inflammation responses, and downregulation of genes involved in DNA damage and repair in both exposures; different expression of genes involved in DNA damage/repair and apoptosis (*tp53*) for starch-coated NPs; upregulation of *cyp1a*; and dysregulation of genes involved in the mitochondrial dysfunction pathway.	[111]
Adults	Fe_2_O_3_: 80-90 nm Fe_3_O_4_: 140-160 nm	28 days	4 and 10 mg/L	Shift in coloration, extravasated blood, and chronic toxicity in the gut.	—	[95]
Adults	23 nm	48 h	20, 50, 100. 140, and 200 mg/kg	Reduction of AChE activity; impaired swimming.	Increased expression of transcriptional *jun*, *caspase-8*, *caspase-9*, *gclc*, *Gpx1a*, *CAT*, *gstp1*, and *sod2*.	[110]

Abbreviations: AChE: acetylcholinesterase; ATP: adenosine-5′-triphosphate; CAT: catalase; *cyp1a*: cytochrome P450 1 A; *gclc*: glutamate-cysteine ligase, catalytic subunit*; Gpx:* glutathione peroxidase; *GST:* glutathione transferase; *HIF1*: hypoxia-inducible factor 1; IO NPs: iron oxide nanoparticles; LC50: 50% of lethal concentration; NO: nitric oxygen; PC: pyruvate carboxylase; ROS: reactivity oxygen species; SOD: superoxide dismutase; *tp53*: tumor protein 53.

**Table 4:** Impact of ZnO NPs on zebrafish.

Stage	NPs diameter	Treatment time	Tested concentrations	General toxicity response	Specific ROS responses	Reference
Embryos	20 nm	96 h	0.1, 0.5, 1, 5, 10, and 50 mg/L	Significant decrease of survival rate; delay in hatching rate dose-dependent; 96 h LC50 = 1.793 mg/L; and several abnormalities (body accusation and pericardial edema).	—	[34]
Embryos	20 nm	96 hpf	0.1, 0.5, 1, 5, 10, 50, and 100 mg/L	Decrease of survival rate; delay in hatching rate; and incidence of pericardial edema dose-dependent.	Increase in ROS production; low levels of *Gstp2* and *Nqo1* expressions; and downfall in counteracting the ROS by oxidative stress responses.	[138]
Embryos	<100 nm	144 hpf	1, 5, 10, 20, 50, and 100 mg/L	No effect bin the survival rate; important decrease in the hatching rate; and different malformations (spinal curvature and hyperemia).	Important elevation in the SOD activity and MDA levels in a dose-dependent way; decrease in CAT activity; high levels of ROS; DNA damage only at the highest concentration tested; and important downregulation in *Bcl-2*, *Nqo1,* and *Gstp2* transcriptions and upregulation in *Ucp-2* level.	[18]
Embryos	30 nm	96 hpf	1, 5, 10, 25, 50, and 100 mg/L	Decrease in survival rate and increase in hatching rate dose-dependent; severe decrease in body length.	—	[18]
Embryos	<100 nm	96 hpf	1, 5, 10, 20, 50, and 100 mg/L	—	Increase in the lipid peroxidation and SOD activity; upregulation in the expression of the *ppaα* and *sod1*; downregulation of *cat*; altered expression of antiapoptotic genes (*bcl-2*) and proapoptotic (*bax*, *puma*, and *apaf-1*; upregulation of *p53* gene, with overexpression of its protein; and increase in the activity of caspase-3 and caspase-9.	[116]
Embryos	9.4 nm	96 hpf	0.2, 1, and 5 mg/L/	Dramatic delay in hatching.	Upregulation of the cat and Cu/Zn-sod transcripts in embryos and downregulation in eleuthero; important upregulation of Mt2<; different expression of mRNA of *IL*-1*β*, *TNFα*, and proinflammatory cytokines in eleuthero-embryos in comparison to embryos; alteration in the jun proto-oncogene (*c-jun*) embryos treated with high concentration; and perturbation in antiviral and immune-related gene Myxovirus resistance A.	[131]
Embryos	50–70 nm	144 hpf	0.1, 0.5, 1, 5, and 10 mg/L	Significant delay in hatching for ZnO NPs and Zn ions; no significant difference in cotreatment with ZnO NPs and NAC; and increased rates of delay in hatching in cotreatment with BSO.	ROS generation; cotreatment with BSO: lower production of GSH.	[132]
Embryos	Nanospheres: 27 nm; nanosticks: 32×81 nmM; and SMPs: 202 nm	120 hpf	2, 4, 8, 16, and 32 mg Zn/L	LC50 for Zn^2+^ =7.9 mg Zn/L, LC50 ZnO SMPs =10.0 mg Zn/L LC50 nanosticks =7.1 Zn/L LC50 nanospheres =11.9 mg Zn/L, respectively; higher toxicity of Zn ions in comparison to the different shaped NPs; and decrease of hatching rate dose-dependent in the embryos treated with all the different kind of nanoparticles and sulfate, strongest delay in samples exposed to nanosticks. Decrease dose-dependent of swimming activity; nanosticks more toxic than the other NPs.	—	[133]
Embryos	5, 10, 15, 26, 34, 62, and 70 nm	120 hpf	0.016 to 250 mg/L	Significant mortality at 24 hpf for all the coated NPs; no alteration in mortality with bare nanoparticles.	—	[134]
Embryos	20-30 nm	96 hpf	0.01, 0.1, 1, and 10 mg/L	Higher mortality rate by ZnO NPs than ZnSO_4_; LC25 for ZnO NPs =2.64 mg/L; LC25 for ZnSO_4_ = 7.75 mg/L; and significant embryonic malformations after both treatments (tail malformation, pericardial edema, and yolk sac edema).	Downregulation of *ogfrl2* and *intl2* transcripts; upregulation of *cyb5d1*.	[17]
Embryos	<100 nm	48 h	10, 30, 60, 90, or 120 mg/L	Delay in hatching, increased heart rate, pericardial edema, hyperemia, yolk sac edema, spinal curvature, tail deformities, and swim bladder abnormalities.	SOD increased activity; *sod1* upregulation; CAT downregulation; increased MDA levels; and increased production of ROS.	[116]
Embryos	40 nm	96 hpf	12.5, 25, 50 mg/L; PFOS (0, 0.4, 0.8, and 1.6 mg/L); and PFOS plus ZnO-NPs (0.4 + 12.5, 0.8 + 25, and 1.6 +50 mg/L	—	Significant increase of SOD activity as well as the activity of glutathione peroxidase; decrease dose-dependent of CAT activity: excess of ROD; increase of MDA level; upregulation of Bax; downregulation of *Bcl-2*; and no changes in the activities of the caspase-3 and caspase-9 (apart at 50 mg/L).	[124]
Embryos	300 nm	72 h, 96 h	10-100 ppm	Mitigate effects on the toxicity of ZnO NPs induced organic matter.	—	[4]
Embryos, adults	PEG =2588 nm PVA =58 nm PVP =60 Bare =69 nm	Embryos: 72 h Adults: 96 h	Embryos: 0.001-100 mg/L	Embryos: morphological defects (yolk sac edema, notochord bending, and egg coagulation) for bare and the capped NPs; rates of toxicity after treatment with 10 mg/L were 38.67%, 28.49%, 95.46%, and 89.32% for PEG-, PVA-, and PVP-capped and bare ZnO NPs, respectively; toxicity of bulk ZnO < ZnO-PVA < ZnO-PEG < ZnO NPs < ZnO-PVP. Adults: LC50 of bulk ZnO =3239 mg/L; LC50 ZnO-PEG =6.44 mg/L; LC50 ZnO-PVA =9.40 mg/L; LC50 ZnO-PVP =3.77 mg/L; LC50 ZnO NPs =20.72 mg/L; and different morphological alterations; severe damages to the gill tissues (secondary lamellar structure alterations, necrosis, desquamation, acute cellular swelling, aneurysm, and lamellar disorganization).	—	[16]
Adults	30 nm	96 h	2, 5, 10, 30, and 50 mg/L	Toxicity dose-dependent; 100% of mortality at 30 mg/mL of ZnO NPs and bulk ZnO; and LC50 = 4.92 mg/L and 3.31 mg/L, for the ZnO NPs and bulk ZnO, respectively.	Increase of SOD activity SOD in the gut; reduction of CAT activity in the liver; increase of CAT activity in gut and gills (only slightly); decrease of GSH in the liver; MDA levels higher in the case of liver; and injuries in the gill tissues with shrinkage of the cells, loss of the cytoplasm, and abnormalities in the nuclei shapes.	[9]

Abbreviations: *Bax*: BCL2-associated X, apoptosis regulator; *bcl2*: B-cell lymphoma 2; BSO: buthionine sulfoximine; CAT: catalase; *cyb5d1*: cytochrome b5 domain containing 1; GSH: glutathione; Gstp2: glutathione S-transferase P 2; hpf: hours post fertilization; *IL-1β:* interleukin-1*β; intl2*: Intelectin 2; LC50: 50% of lethal concentration; MDA: malondialdehyde; Mt2: metalloprotein 2; NAC: N-acetyl cysteine; *Nqo1*: NAD(P)H:quinone oxidoreductase; *ogfrl*2: opioid growth factor receptor-like; PEG: polyethylene glycol; PVA: polyvinyl alcohol; PVP: polyvinylpyrrolidone; PFOS: perfluoro octane sulfonate; ROS: reactive oxygen species; SMPs: submicron particles; *TNFα*: tumor necrosis factor-*α*; *Ucp-2*: uncoupling protein 2.

## Data Availability

All data are included in the manuscript.
